# T-bet controls intestinal mucosa immune responses via repression of type 2 innate lymphoid cell function

**DOI:** 10.1038/s41385-018-0092-6

**Published:** 2018-10-24

**Authors:** N. Garrido-Mesa, J-H. Schroeder, E. Stolarczyk, A. L. Gallagher, J. W. Lo, C. Bailey, L. Campbell, V. Sexl, T. T. MacDonald, J. K. Howard, R. K. Grencis, N. Powell, G. M. Lord

**Affiliations:** 1 School of Immunology and Microbial Sciences, King’s College London, London, SE1 9RT UK; 2 Division of Diabetes and Nutritional Sciences, King’s College London, London, SE1 9NH UK; 3 Faculty of Biology, Medicine and Health, University of Manchester, Manchester, M13 9PT UK; 4 Institute of Pharmacology and Toxicology, University of Veterinary Medicine, Vienna, 1210 Austria; 5 Centre for Immunobiology, Blizard Institute, Barts & The London School of Medicine and Dentistry, Queen Mary University of London, London, E1 2AT UK; 6 Department of Inflammation Biology, Centre for Inflammation Biology & Cancer Immunology, School of Immunology & Microbial Sciences, King’s College London, London, UK; 7 Present Address: School of Health, Sports and Bioscience, University of East London, London, E15 4LZ UK; 8 Present Address: Division of Diabetes, Endocrinology and Metabolism, Imperial College London, London, W12 0NN UK

## Abstract

Innate lymphoid cells (ILCs) play an important role in regulating immune responses at mucosal surfaces. The transcription factor T-bet is crucial for the function of ILC1s and NCR^+^ ILC3s and constitutive deletion of T-bet prevents the development of these subsets. Lack of T-bet in the absence of an adaptive immune system causes microbiota-dependent colitis to occur due to aberrant ILC3 responses. Thus, T-bet expression in the innate immune system has been considered to dampen pathogenic immune responses. Here, we show that T-bet plays an unexpected role in negatively regulating innate type 2 responses, in the context of an otherwise intact immune system. Selective loss of T-bet in ILCs leads to the expansion and increased activity of ILC2s, which has a functionally important impact on mucosal immunity, including enhanced protection from *Trichinella spiralis* infection and inflammatory colitis. Mechanistically, we show that T-bet controls the intestinal ILC pool through regulation of IL-7 receptor signalling. These data demonstrate that T-bet expression in ILCs acts as the key transcriptional checkpoint in regulating pathogenic vs. protective mucosal immune responses, which has significant implications for the understanding of the pathogenesis of inflammatory bowel diseases and intestinal infections.

## Introduction

Innate lymphoid cells (ILCs) play a crucial role in early mucosal immune defence, inflammation and tissue remodelling.^1^ Based on the transcription factors that govern their differentiation, function and signature cytokine production, mature IL-7Rα^+^ ILCs can be classified into three groups: ILC1s, which express T-bet and produce IFNγ and TNFα; ILC2s that express high levels of GATA-3 and produce type 2 cytokines; and ILC3s that express ROR*γ*t, produce IL-22 and IL-17,^1^ and can be further subdivided based on the expression of the chemokine receptor CCR6 and the natural cytotoxicity receptor NKp46 or NCR, encoded by the gene *Ncr1*.^2^

In addition to ILC1s, T-bet is co-expressed with NKp46 in NCR^+^ ILC3s (in which it is co-expressed with RORγt) and cNK cells.^2,3^ T-bet is essential for ILC development, as germline deletion of T-bet results in a complete loss of NCR^+^ ILCs,^2,4^ and it is also involved in NCR^+^ ILC function. The first description of this came from studies in the TRUC mouse (*T-bet*^*−/−*^ x *RAG2*^*−/−*^ ulcerative colitis), which develop spontaneous colitis dependent on IL-17-producing ILC3s.^5^ Furthermore, within the CCR6^−^ ILC3 subset, T-bet expression is required for CCR6^-/low^ ILC3s differentiation into NCR^+^ ILC3s and subsequent IFNγ production.^2,6,7^T-bet is also induced in human and murine ILC2s, resulting in the production of IFNγ.^8–11^ Among several cytokines, IL-12 and IL-18 appear to be the main driver of these effects, however their pathophysiological relevance is still unknown.

The absence of ILC1s in *T-bet*^*−/−*^ mice is linked to increased susceptibility to enteric infections.^2,6,12–14^ We have previously reported the phenotype of TRUC mice that develop spontaneous colitis, which is dependent on IL-17-producing CCR6^+^ ILC3s in the absence of adaptive immunity.^5^ Increased frequency of inflammatory ILC1s has also been found in inflamed intestine from Crohn’s disease patients.^6,13,15^ However, whether T-bet expression in ILCs drives protective or pathogenic mucosal immune responses in the presence of an intact immune system still needs to be elucidated. Importantly, we have recently shown that alterations in T-bet binding are critical determining factors in driving mucosal inflammatory diseases in humans.^16^

Here, we define a previously unrecognised role for T-bet in ILC2 function and its significance in the control of helminth infection and the pathogenesis of dextran sodium sulphate (DSS)-induced colitis. In the presence of an intact immune system, specific targeting of T-bet in ILCs results in the development of protective intestinal ILC2 responses. Crucially, T-bet regulates responsiveness of the intestinal ILC pool to IL-7 signalling. Therefore, we propose that T-bet acts as a key molecular regulator, controlling both pathogenic and protective immune responses in the intestine in a context-dependent manner.

## Results

### ILC2s are expanded in immunocompetent T-bet-deficient mice

**Fig. 1:**
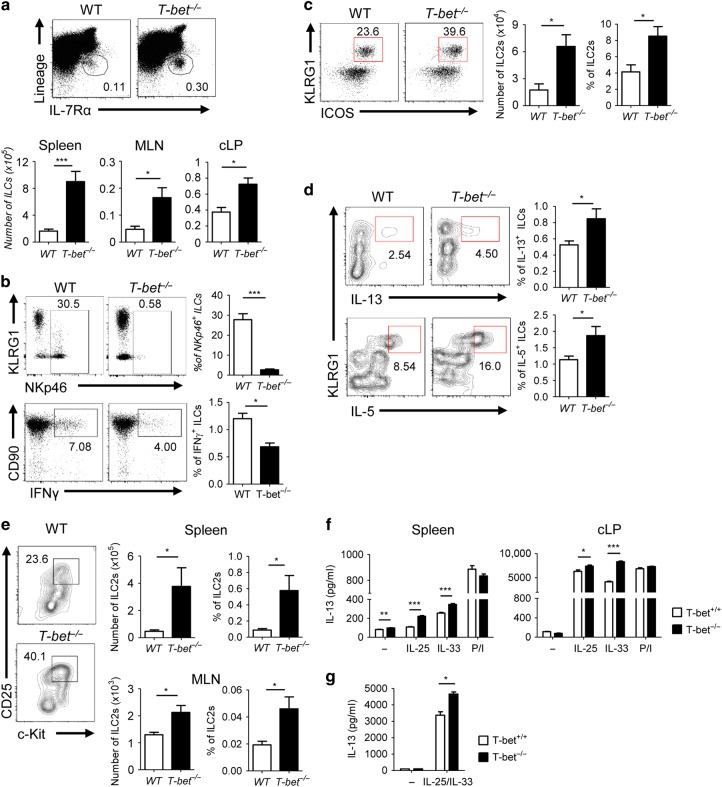
ILC2s are expanded in immunocompetent T-bet-deficient mice. **a**–**g** Flow cytometry analysis of the different ILC populations in WT and *T-bet*^*−/−*^ mice showing: **a** Representative plots showing the ILC population within CD45^+^ cells in the spleen. Absolute cell numbers of ILCs in the indicated tissues; **b** Representative plots and percentage of NKp46^+^ cells within the ILC population and IFNγ-producing ILCs within the CD45^+^ live cell population in the cLP; **c** Representative plots showing the ILC2 population within cLP ILCs. Absolute cell numbers of ILC2s and percentage of ILC2s within the CD45^+^ live cell population in the cLP; **d** Representative plots showing IL-13 and IL-5 cytokine expression by KLRG1^+^ ILCs from the cLP. Percentage of IL-13^+^ and IL-5^+^ ILCs within the CD45^+^ live cell population in the cLP; **e** Representative plots showing the ILC2 population within splenic ILCs. Absolute cell numbers of ILC2s and percentage of ILC2s within the CD45^+^ live cell population in the indicated tissues. ILCs are defined as CD45^+^Lin^-^IL-7Rα^+^ cells; ILC2s are defined as CD45^+^Lin^-^IL-7Rα^+^ICOS^+^KLRG1^+^ (cLP) and as CD45^+^Lin^−^IL-7Rα^+^CD25^+^c-Kit^+^ (spleen and MLN) cells. **f-g** IL-13 concentration in the supernatants of cultured: **f** T-cells-depleted leucocytes from the spleen and cLP and **g** FACS-sorted ILC2s from the cLP of WT and *T-bet*^*−/−*^ mice, unstimulated (-) or stimulated with IL-25 or/and IL-33 (50 ng/ml, 24 h), or PMA (50 ng/ml) and ionomycin (1 μg/ml). ILC2s were FACS-sorted as CD45^+^Lin^-^IL-7Rα^+^ICOS^+^KLRG1^+^ cells. Data are expressed as mean ± SEM and are representative of at least three independent experiments (*n* = 3). ns: non-significant; *p < 0.05; **p < 0.01; ***p < 0.001. See also Supplemental Figs. 1–4

We found markedly increased numbers of ILCs in the spleen, mesenteric lymph nodes (MLN) and colon lamina propria (cLP) of *T-bet*^*−/−*^mice compared to wild type (WT) mice (Fig. 1a). As previously reported, NCR^+^ ILCs were virtually absent in *T-bet*^*−/−*^mice and IFNγ production by ILCs was decreased^4^ (Fig. 1b), similar to the situation with T-bet-deficient NK and T cells (Supplemental Fig. 1). To date, there are no data informing how T-bet might impact ILC2 numbers in the gut. Strikingly, the KLRG1^+^ICOS^+^ ILC2s were markedly expanded in the cLP of *T-bet*^*−/−*^mice (Fig. 1c). Likewise, the proportion of IL-5 and IL-13-producing ILC2s were also significantly increased in the cLP of *T-bet*^*−/−*^mice (Fig. 1d). This ILC2 expansion was also observed in the spleen and MLN of *T-bet*^*−/−*^mice where the number of CD25^+^cKit^+^ ILC2s was increased by up to 8- and 3-fold, respectively (Fig. 1e) (Supplemental Fig. 2). This phenotype was independent of the reduction in IFNγ production that characterises *T-bet*^*−/−*^ mice, as no differences were observed in ILC2 numbers between WT and *Ifng*^*−/−*^ mice (Supplemental Fig. 3). To further investigate the functionality of ILC2s in the absence of T-bet, T-cell-depleted leucocytes from the spleen and the cLP of T-bet-deficient and sufficient mice were stimulated overnight with IL-25 or IL-33, two potent stimulators of ILC2s,^17,18^ which resulted in higher levels of IL-13 detected in the culture supernatants of T-bet-deficient cells (Fig. 1f). This observation was in line with greater abundance of IL-13-expressing cells among T-bet-deficient ILC2s upon a 4-hour in vitro stimulation with IL-25 or IL-33. IL-25 and IL-33 receptor expression in ILC2s from *T-bet*^*−/−*^ mice was similar to that observed in WT ILC2s (Supplemental Fig. 4). When *T-bet*^*−/−*^and *T-bet*^*+/+*^ cultures where activated with PMA and ionomycin overnight no differences in IL-13 levels in the culture supernatants were observed, suggesting that IL-25/IL-33 specific responsive cells were responsible for the differential IL-13 production detected. To further address whether the enhanced expression of IL-13 in this model was due to increased ILC2s numbers within the ILC population or due to a higher ILC2 per cell responsiveness, ILC2s from *T-bet*^*−/−*^and WT mice were FACS-sorted and equal numbers of cells were stimulated in vitro with IL-25 and IL-33. IL-13 levels in culture supernatants were significantly higher in ILC2 cultures from *T-bet*^*−/−*^mice than from T-bet-sufficient animals (Fig. 1g). Therefore, we concluded that T-bet deficiency leads to the expansion of ILC2s and enhances ILC2 cytokine production in response to IL-25 and IL-33.

### ILC2 expansion also occurs in the context of T-bet deficiency in the absence of an adaptive immune system

**Fig. 2:**
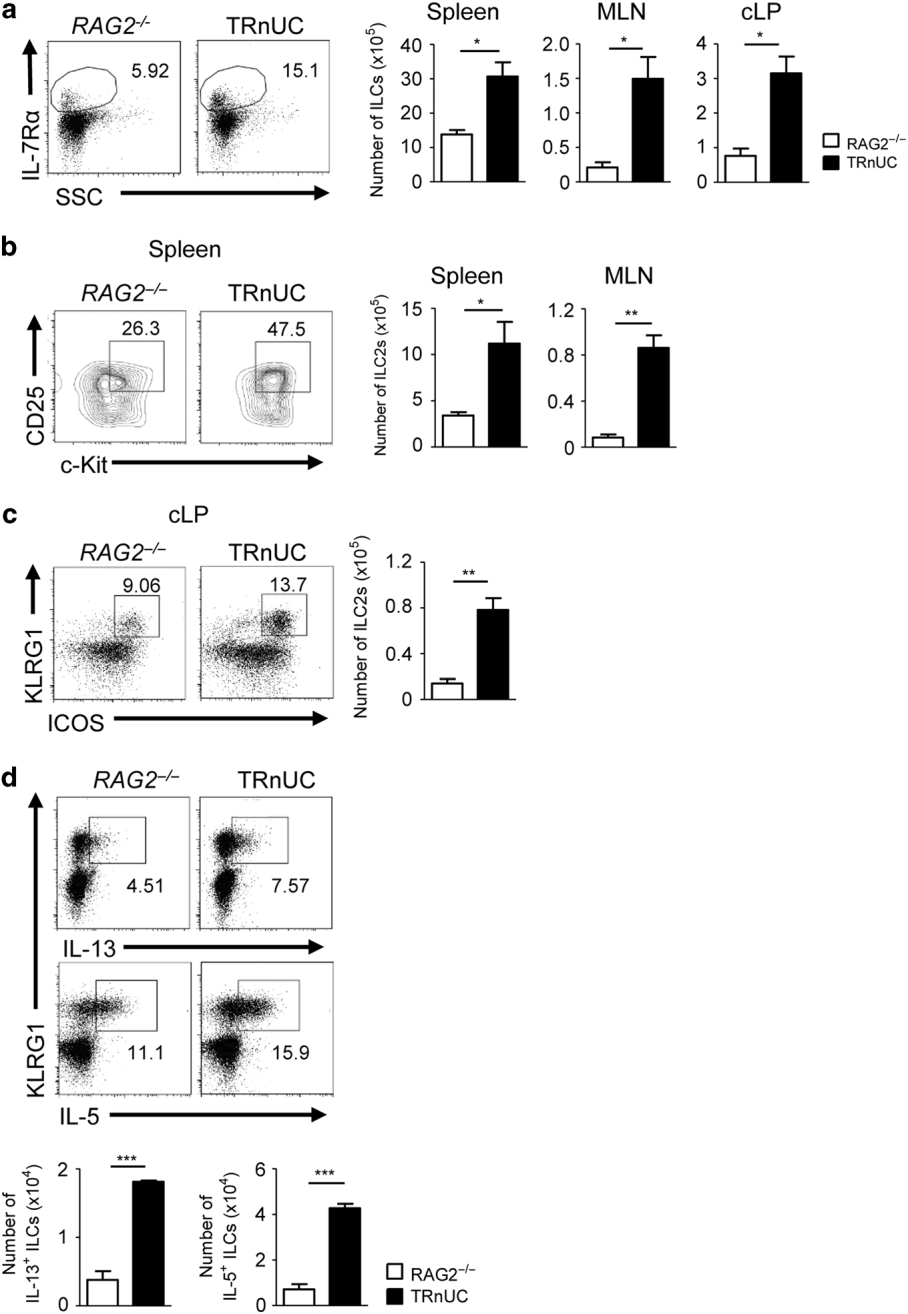
ILC2 expansion also occurs in *T-bet*^*−/−*^ x *RAG2*^*−/−*^mice. Flow cytometry analysis of the different ILC populations in *RAG2*^*−/−*^and TRnUC mice showing: **a** Representative plots showing the ILC population within CD45^+^ cells from the spleen. Absolute numbers of ILCs in the indicated tissues; **b** Representative plots showing the ILC2 population within ILCs from the spleen and absolute numbers of ILC2s in the spleen and MLN; **c** Representative plots showing the ILC2 population within ILCs from the cLP and absolute numbers of ILC2s in the cLP; **d** Representative plots showing IL-5 and IL-13 expression by KLRG1^+^ ILCs and numbers of IL-5 and IL-13-producing ILCs in the cLP. Data are expressed as mean ± SEM and are representative of at least three independent experiments (*n* = 3). *p < 0.05; **p < 0.01; ***p < 0.001. ILCs are defined as CD45^+^IL-7Rα^+^ cells and ILC2s as CD45^+^IL-7Rα^+^cKit^+^CD25^+^ (spleen and MLN) and CD45^+^IL-7Rα^+^KLRG1^+^ICOS^+^ (cLP) cells

T-bet deficiency in the innate immune system has been previously linked with aberrant IL-17 production by ILCs, as seen in the TRUC model, a colony of *T-bet*^*−/−*^ x *RAG2*^*−/−*^ that develops microbiota-dependent colitis in response to *Helicobacter typhlonius*.^5^ We have now taken advantage of a colitis-free colony of *T-bet*^*−/−*^ x *RAG2*^*−/−*^ non*-*ulcerative colitis mice or TRnUC mice and evaluated ILC2 responses in this context. Higher numbers of ILCs and ILC2s were also found in the spleen, MLN and cLP of TRnUC mice when compared with *RAG2*^*−/−*^ mice (Fig. 2a–c), and the numbers of IL-5 and IL-13-producing ILCs in the cLP was also substantially increased (Fig. 2d).

### In vitro targeting of T-bet in ILCs promotes the ILC2 phenotype

**Fig. 3:**
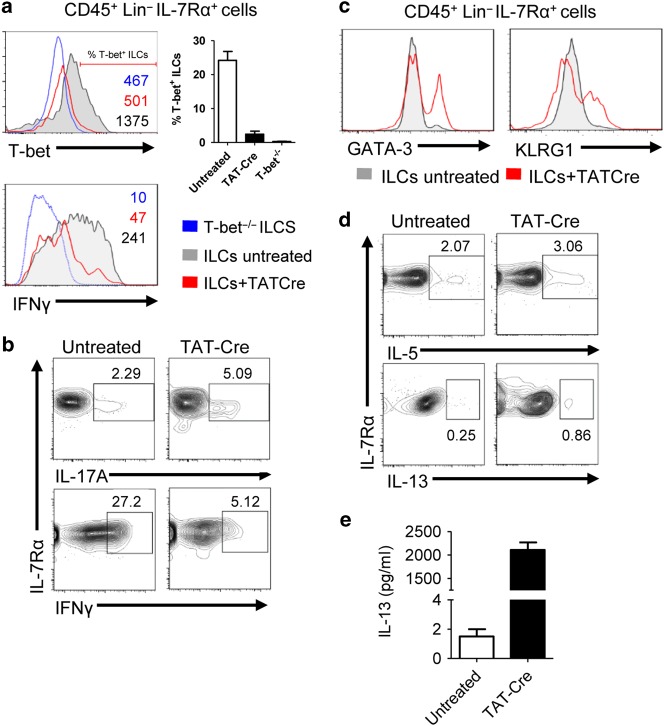
In vitro targeting of T-bet in ILCs promotes the ILC2 phenotype. **a**–**d** Representative flow cytometry plots and histograms showing: **a** T-bet expression and IFNγ production (numbers indicate mean fluorescent intensity (MFI)) and percentage of T-bet^+^ ILCs; **b** IL-17A and IFNγ production (numbers indicate percentage of cells); **c** GATA-3 and KLRG1 expression and **d** IL-5 and IL-13 production (numbers indicate percentage of cells); **e** Concentration of IL-13 in the culture supernatants. FACS-sorted cLP ILCs (CD45^+^Lin^−^IL-7Rα^+^cells) from *T-bet*^*fl/fl*^ mice were treated with TAT-Cre recombinase (100 μg/ml) and maintained in culture with IL-2 (100 UI/ml) and IL-7 (10 ng/ml) for a week. Intracellular cytokine staining was performed after PMA and ionomycin stimulation. For IL-13 determination in the culture supernatant cells were previously stimulated with IL-25 and IL-33 (50 ng/ml) for 24 h. Data are expressed as mean ± SEM and are representative of at least three independent experiments (*n* = 3)

The direct role of T-bet in ILC function was also evidenced in T-bet deletion studies in ex vivo purified ILCs. In vitro treatment of ILCs from *T-bet*^*fl/f*^ mice^19^ with TAT-Cre recombinase caused a reduction in T-bet expression to levels close to those detected in ILCs from *T-bet*^*−/−*^ mice (Fig. 3a), and consequently their ability to produce IFNγ was also reduced, while they produced more IL-17A (Fig. 3a, b). Interestingly, this was accompanied by an increase in GATA-3 and KLRG1 expression (Fig. 3c) and a higher production of IL-5 and IL-13, as detected by flow cytometry and in the culture supernatants of cells exposed to the Cre-recombinase (Fig. 3d, e). These findings are consistent with a transition towards an ILC2 phenotype and demonstrate a novel role for T-bet in ILCs, controlling the stability of ILC1s while repressing ILC2 activity and development.

### De-repression of IL-7Rα in *T-bet*^*−/−*^ ILCs stabilises IL-7 signalling

**Fig. 4:**
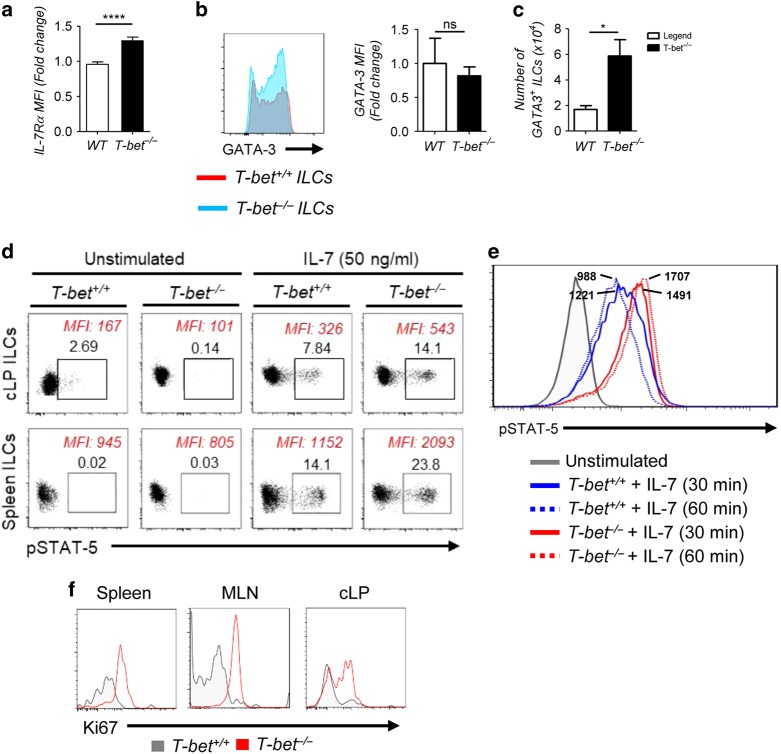
T-bet de-repression of IL-7Rα in ILCs stabilises IL-7 signalling and STAT-5 phosphorylation. **a** Density of IL-7Rα expression in ILCs from the cLP of WT vs. *T-bet*^*−/−*^ mice measured by flow cytometry. Fold change expressed as mean ± SEM vs. WT group. **b** GATA-3 expression levels in ILCs from WT *vs T-bet*^*−/−*^ mice. **c** Absolute cell numbers of GATA-3^+^ ILCs in the cLP of WT and *T-bet*^*−/−*^ mice. **d**–**e** Representative flow cytometry analysis of the phosphorylation of STAT-5 in ILCs from the spleen (**d**) and cLP (**d**–**e**) of WT and *T-bet*^*−/−*^mice after stimulation with: **d** IL-7 (50 ng/ml, 60 min) and **e** IL-7 (20 ng/ml, 30 or 60 min). Numbers indicate the median fluorescence intensity (MFI). **f** Representative flow cytometry analysis of Ki67 expression in ILC2s from the spleen, MLN and cLP of WT and *T-bet*^*−/−*^mice. Data are expressed as mean ± SEM and are representative of at least three independent experiments (*n* = 3).*p < 0.05; ****p < 0.0001. ILCs were defined as CD45^+^Lin^-^IL-7Rα^+^ and ILC2s as CD45^+^Lin^-^IL-7Rα^+^ICOS^+^cKit^+^CD25^+^ (spleen and MLN) and CD45^+^Lin^−^IL-7Rα^+^KLRG1^+^ICOS^+^ (cLP) cells. See also Supplemental Figs. 5 and 6

IL-7 signalling has a critical role in ILC development and function^20^ and decreased IL-7Rα expression by ILC2s in response to retinoid acid has been related to reduced proliferation and tissue accumulation of ILC2s.^21^ We observed that IL-7Rα expression was significantly higher in ILCs from *T-bet*^*−/−*^ than from WT mice (Fig. 4a). Interestingly, while GATA-3 has previously been described to positively regulate IL-7Rα expression in ILC3s,^22^ we observed the levels of GATA-3 expression in total ILCs from *T-bet*^*−/−*^ and WT mice to be found equivalent (Fig. 4b), even though higher numbers of GATA-3^+^ ILCs were found in *T-bet*^*−/−*^ mice (Fig. 4c). IL-7 signalling activates STAT-5^23^ and in T-cells, inducing GATA-3 and IL-13 expression and driving Th2 differentiation.^24,25^ Considering that GATA-3 is also critical for ILC2 maintenance and IL-13 production,^26–31^ ILCs may also require an IL-7-driven STAT-5 signal to induce GATA-3 and ILC2 differentiation. Interestingly, after IL-7 stimulation, we found increased phosphorylation of STAT-5 in ILCs from *T-bet*^*−/−*^ mice in comparison with T-bet-sufficient ILCs (Fig. 4d, e), while no activation of STAT-3 or STAT-4 was induced (Supplemental Fig. 5). Considering the role of IL-2 in ILC biology, STAT-5 activation was also analysed in ILCs upon stimulation with IL-2 and no significant differences were observed between T-bet- sufficient and T-bet-deficient ILCs (Supplemental Fig. 6A). In addition, the expression of the IL-2 receptor CD25 was comparable in ILC2s from WT and T-bet^−/−^ mice (Supplemental Fig. 6B). Of note, higher frequencies of Ki67-expressing ILC2s were observed in ILC cultures from *T-bet*^*−/−*^spleen, MLN and cLP (Fig. 4f). Thus, in the absence of T-bet, de-repression of IL-7Rα in ILCs conferred enhanced IL-7 signalling capacity, driving STAT-5 phosphorylation and potentially mediating the expansion and increased cytokine production activity of ILC2s.

### Development of an in vivo model of selective deletion of T-bet in ILCs

We have shown that deletion of T-bet triggers ILC2s expansion and activity both in vivo and in vitro. However, T-bet deficiency in other non-ILC immune cells or the lack of NCR^+^ ILCs in *T-bet*^*−/−*^ mice per se, could potentially impact on this phenotype. In addition, previous reports have suggested that ILC homoeostasis is altered in models lacking adaptive immunity^32,33^ and it has been shown that ILCs and T cells compete for IL-7^34^ suggesting that ILCs may expand in models lacking adaptive immunity as a result of the higher availability of IL-7. Therefore, we sought to develop a model of selective deletion of T-bet in ILCs in an otherwise intact immune system, which would allow for a more accurate in vivo interrogation of the role of T-bet in ILC homoeostasis and function.

T-bet expression in ILCs has been reported to be linked to the expression of the natural cytotoxicity receptor NKp46 (encoded by *Ncr1*) (Supplemental Fig. 7), expressed in cNK cells, ILC1s and a subset of ILC3s.^3^ Therefore, to selectively target T-bet in ILCs, *T-bet*^*fl/fl*^ mice^19^ were crossed with the *Ncr1*-iCre^Tg^ transgenic mouse,^35^ which expresses the Cre-recombinase under the control of the *Ncr1* promoter, a strategy previously used to delete GATA-3 specifically in ILCs.^36^

**Fig. 5:**
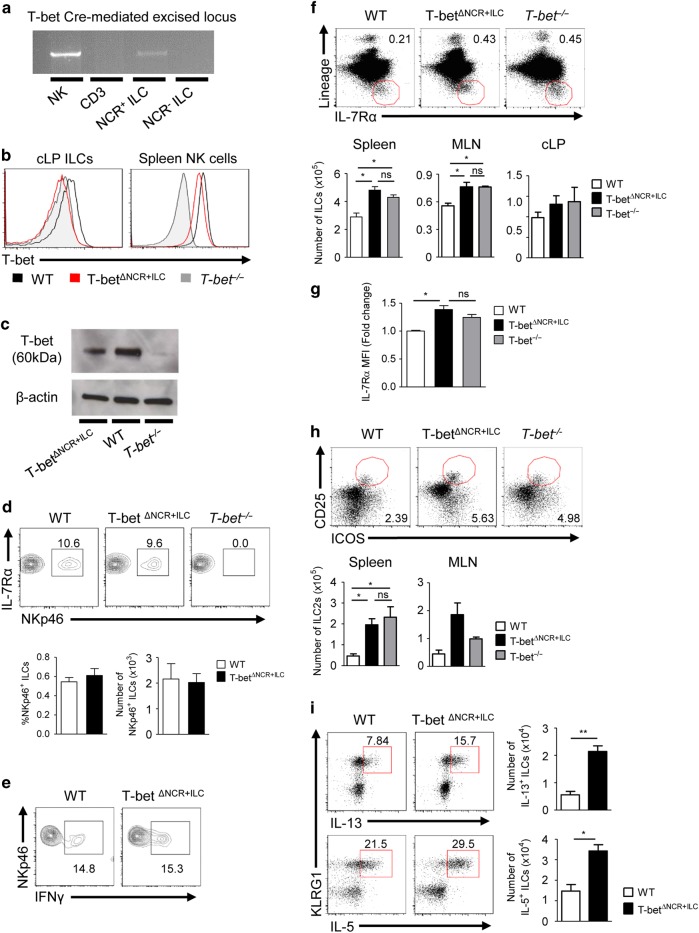
Selective deletion of T-bet in ILCs in vivo. **a** Agarose gel electrophoresis (1.5%) of PCR products following specific genomic DNA PCR for the Cre-mediated excised locus of T-bet in FACS-sorted NK cells (CD45^+^IL-7Rα^−^NK1.1^+^NKp46^+^), T-cells (CD3^+^), NCR^+^ ILCs (CD3^-^IL-7Rα^+^NKp46^+^) and NCR^−^ ILCs (CD3^−^IL-7Rα^+^NKp46^−^) from T-bet^ΔNCR+ILC^ mice. **b** Representative histograms showing intracellular staining for T-bet in ILCs from the cLP and in NK cells from the spleen of WT, T-bet^ΔNCR+ILC^ and *T-bet*^*−/−*^mice. **c** Analysis of T-bet expression by western blot in FACS-sorted NK cells from the spleen. **d** Representative flow cytometry plots showing the percentage of NCR^+^ ILCs and percentage within the CD45^+^ live cell population and absolute numbers of NCR^+^ ILCs in the cLP of WT, T-bet^ΔNCR+ILC^ and *T-bet*^*−/−*^mice. **e** Representative flow cytometry plots showing IFNγ production by NCR^+^ ILCs in the cLP of WT and T-bet^ΔNCR+ILC^ mice. **f** Representative flow cytometry plots showing the percentages of ILCs in the spleen and absolute numbers of ILCs in the indicated tissues. **g** Density of IL-7Rα expression in ILCs from WT vs. T-bet^ΔNCR+ILC^ mice measured by flow cytometry. Fold change expressed as mean ± SEM vs. WT group. **h** Representative flow cytometry plots showing the percentages of ILC2s within the CD45^+^ live cell population in the spleen and absolute cell numbers of ILC2s in the indicated tissues of WT, T-bet^ΔNCR+ILC^ and *T-bet*^*−/−*^mice. **i** Representative flow cytometry analysis of IL-5 and IL-13 expression by ILCs and absolute cell numbers of IL-5^+^ and IL-13^+^ ILCs in the cLP of WT and T-bet^ΔNCR+ILC^ mice. Data are expressed as mean ± SEM and are representative of at least three independent experiments (*n* = 3). ns: non-significant; *p < 0.05; **p < 0.01. ILCs were defined as CD45^+^Lin^−^IL-7Rα^+^ cells and ILC2s were defined as CD45^+^Lin^–^IL-7Rα^+^CD25^+^ICOS^+^ (spleen and MLN) and CD45^+^Lin^–^IL-7Rα^+^ICOS^+^KLRG1^+^ (cLP) cells. See also Supplemental Figs. 7–9

Genomic PCR in FACS-sorted cells evidenced the presence of the T-bet-recombined locus in cNK cells and NKp46-expressing or NCR^+^ ILCs, but not in NCR^−^ILCs or CD3^+^ cells (Fig. 5a). Importantly, no reduction in T-bet expression was observed in NKp46^+^ CD3^+^ cells and the ability of naïve CD4^+^ T-cells to differentiate into Th1 or Th2 cells was also unaffected (Supplemental Fig. 8). In contrast to the work of others using a different genetic approach to drive Cre-recombinase,^37^ efficient deletion of T-bet in ILCs was achieved in this model. T-bet expression in ILCs from the *Ncr1*-iCre^Tg^ x *T-bet*^*fl/fl*^ mouse was equivalent to that of ILCs from *T-bet*^*−/−*^ mice (Fig. 5b, left). Although the T-bet recombined locus was also observed in NK cells, in these cells T-bet expression was only reduced by approximately 25% (Fig. 5b, c), and this had no effect on their maturation or their ability to produce IFNγ (Supplemental Fig. 9), as occurs in germline T-bet deficiency. Notably, and in contrast to previous reports describing the absence of NCR^+^ ILCs in *T-bet*^*−/−*^ mice,^2,4^ we found that selective deletion of T-bet in NCR^+^ ILCs did not affect their development or cytokine-producing ability. NCR^+^ ILCs were found in the cLP of *Ncr1*-iCre^Tg^ x *T-bet*^*fl/fl*^ mice at similar percentages and numbers as in WT littermate controls (Fig. 5d), and their IFNγ producing ability remained unaffected (Fig. 5e). These findings support the *Ncr1*-iCre^Tg^ x *T-bet*^*fl/fl*^ or T-bet^ΔNCR+ILC^ mouse as an in vivo model of selective deletion of T-bet in ILCs in an immunocompetent background.

Importantly, the number of ILCs in the spleen and MLN of T-bet^ΔNCR+ILC^ mice was increased (Fig. 5f) and they expressed higher cell surface IL-7Rα (Fig. 5g), as previously observed in *T-bet*^*−/−*^ mice. Similarly, ILC2s were found to be substantially increased in the spleen and MLN of T-bet^ΔNCR+ILC^ mice (Fig. 5h) and stimulation of cLP leucocytes resulted in higher IL-13 and IL-5 production by ILC2s (Fig. 5i). These results show that in vivo targeting of T-bet in ILCs selectively mediates the expansion of ILC2s, reproducing the findings observed in vitro and establishing a central role for T-bet in the development and function of ILC2s.

### T-bet deficiency enhances the mucosal immune response against intestinal parasites

**Fig. 6:**
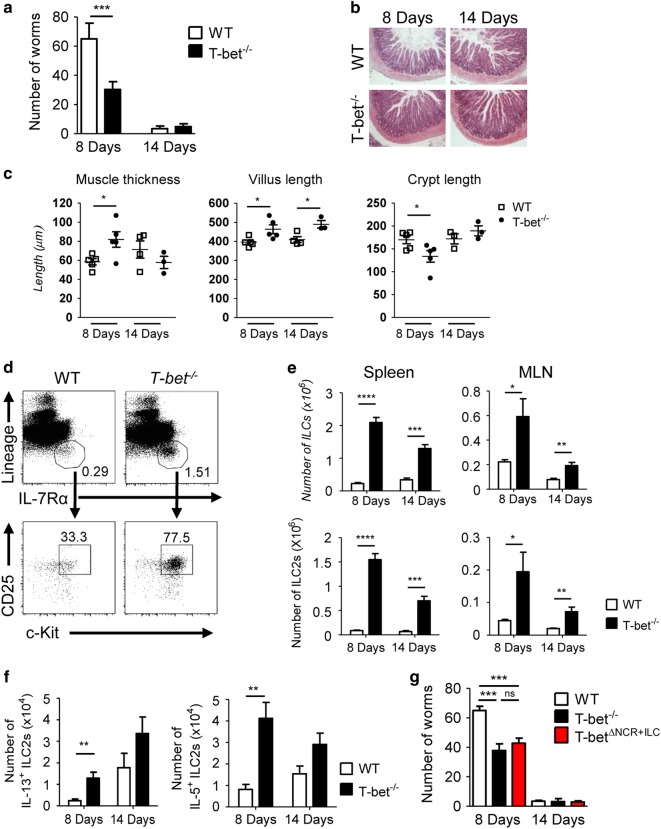
T-bet deficiency enhances the mucosal immune response against intestinal parasites. **a** Number of worms in the SI of WT and *T-bet*^*−/−*^ mice 8 and 14 days after the infection with 300 larvae of *T. spiralis*. **b** Representative H&E-stained histological sections and **c** Quantification of the muscle thickness, villus length and crypt depth in SI histological sections from *T. spiralis* infected mice. **d** Representative flow cytometry analysis of CD45^+^ cells from the spleen of *T. spiralis* infected mice showing percentages of ILCs and of ILC2s in *T-bet*^*−/−*^ mice 8 days after the infection. **e** Absolute cell numbers of ILCs and ILC2s in the indicated tissues, and **f** Absolute cell numbers of IL-5^+^ and IL-13^+^ ILC2s in the spleen of *T. spiralis* infected mice, 8 and 14 days after the infection. **g** Number of worms in the SI of WT, *T-bet*^*−/−*^ and T-bet^ΔNCR+ILC^ mice 8 and 14 days after the infection with 300 larvae of *T. spiralis*. Data are expressed as mean ± SEM and are representative of at least three independent experiments (*n* = 5). ns: non-significant; *p < 0.05; **p < 0.01; ***p < 0.001; ****p < 0.0001. ILCs are defined as CD45^+^Lin^−^IL-7Rα^+^ cells and ILC2s as CD45^+^Lin^−^IL-7Rα^+^CD25^+^c-Kit^+^ (spleen and MLN) and CD45^+^Lin^−^IL-7Rα^+^KLRG1^+^ (cLP) cells

We next studied the functional implications of loss of T-bet in ILCs in the development of mucosal immune responses. ILC2 responses are essential in mediating immunity against intestinal parasites,^17,18,38,39^ and previous studies have shown that T-bet-deficient mice display an accelerated expulsion of *Trichinella spiralis*.^40^ In agreement with this, we observed that *T. spiralis* worm expulsion was enhanced in *T-bet*^*−/−*^ mice compared to WT mice 8 days after the infection, while after 14 days both groups had equally eliminated the worms (Fig. 6a). Haematoxylin and eosin (H&E) histological analysis revealed that at day 8 the small intestine (SI) muscle layer in *T-bet*^*−/−*^ mice was thicker than in WT mice, the villus length was preserved and the depth of the crypts was reduced (Fig. 6b, c). Regarding the ILC population, significantly higher numbers of ILCs and especially ILC2s were found in the spleen and MLN of infected *T-bet*^*−/−*^ mice (Fig. 6d, e) and higher numbers of IL-5 and IL-13-producing ILC2s were evidenced in the spleen of *T-bet*^*−/−*^ mice during the early response to the parasite (Fig. 6f). A link between T-bet-deficient ILCs and accelerated worm expulsion was confirmed in the T-bet^ΔNCR+ILC^ model. Eight days after the infection with *T. spiralis*, the number of worms remaining in the SI of T-bet^ΔNCR+ILC^ mice was significantly lower than in WT mice (Fig. 6g). In fact, worm expulsion in T-bet^ΔNCR+ILC^ mice was as marked as in *T-bet*^*−/−*^ mice, supporting the involvement of T-bet-deficient ILCs in this process.

### T-bet deficiency in ILCs protects from the development of inflammatory colitis

**Fig. 7:**
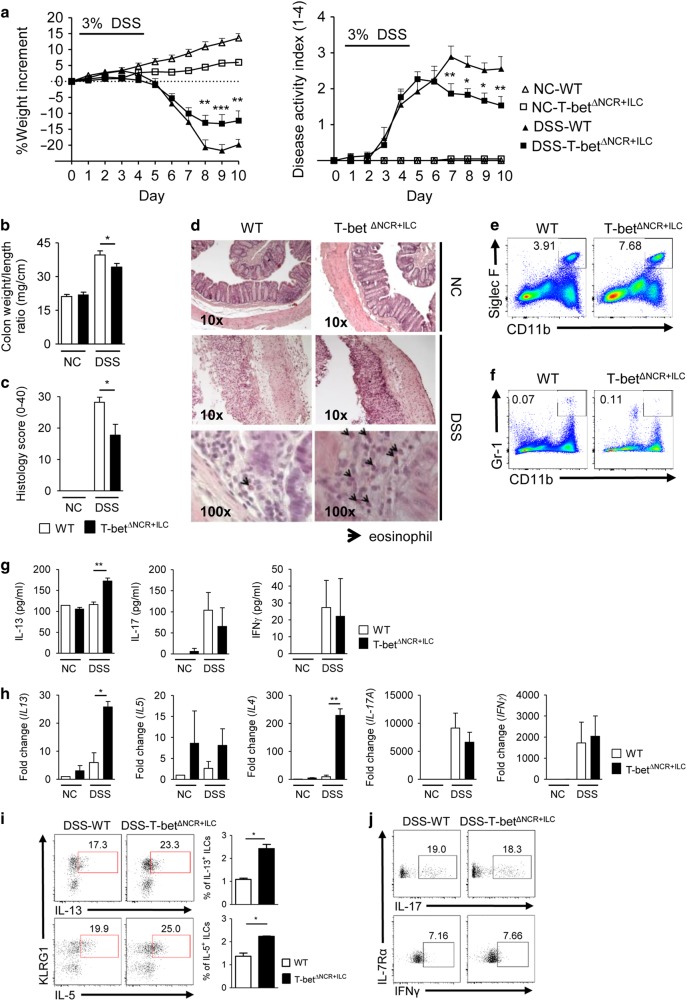
T-bet deficiency in ILCs protects from the development of inflammatory colitis. **a** Weight increment (%) (left panel) and disease activity index (DAI) values (right panel) of NC and DSS WT and T-bet^ΔNCR+ILC^ mice over the 10-day experimental period (*n* = 10). DAI values were calculated based on the criteria proposed previously.^56^ **b** Colon weight/length ratio and **c** Spleen weight of NC and DSS WT and T-bet^ΔNCR+ILC^ mice (*n* = 10). **c** Microscopic damage score assigned to colonic sections according the criteria described in supplemental table (*n* = 10) and **d** Representative H&E-stained colonic sections from NC and DSS WT and T-bet^ΔNCR+ILC^ (arrows indicate eosinophils). **e**, **f** Representative flow cytometry plots showing the presence of **e** SiglecF^+^CD11b^+^ eosinophils and **f** Gr1^+^CD11b^+^ neutrophils, in the cLP of WT and T-bet^ΔNCR+ILC^ mice. **g** Concentration of the indicated cytokines in the culture supernatants of explant colon organ cultures and **h** Real-time PCR measuring the transcripts of the indicated cytokines in the colon of NC and DSS WT and T-bet^ΔNCR+ILC^ mice (*n* = 3). Fold change expressed as mean ± SEM vs. NC WT group. **i**, **j** Representative flow cytometry analysis of intracellular cytokine production showing: **i** IL-5 and IL-13 production and percentages of IL-5^+^ and IL-13^+^ ILCs within the CD45^+^ live cell population (*n* = 3) and **j** IL-17 and IFNγ production by cLP ILCs from DSS WT and T-bet^ΔNCR+ILC^ mice. Data are expressed as mean ± SEM and are representative of at least three independent experiments. *p < 0.05; **p < 0.01; ***p < 0.001. ILCs are defined as CD45^+^Lin^−^IL-7Rα^+^ cells and ILC2s as CD45^+^Lin^−^IL-7Rα^+^CD25^+^c-Kit^+^ (spleen and MLN) and CD45^+^Lin^−^IL-7Rα^+^KLRG1^+^ (cLP) cells. Eosinophils and neutrophils are gated on live CD45^+^ cells. NC and DSS represent non-colitic and DSS-treated mice, respectively. See also Supplemental Fig. 10

To address the functional relevance of these findings in the context of type 1 mucosal inflammation, we induced acute colitis in T-bet^ΔNCR+ILC^ mice with DSS.^41^ It has previously been shown that ILC2s play a protective role in DSS colitis,^42^ therefore, we hypothesised that the expansion of ILC2s observed in mice lacking T-bet in the ILC compartment would be protective from colitis. Interestingly, T-bet^ΔNCR+ILC^ mice developed a milder systemic disease in response to DSS, as evidenced by their significantly reduced weight loss and lower disease activity index (DAI) score in comparison with control mice (Fig. 7a). The colon weight/length ratio in colitic T-bet^ΔNCR+ILC^ mice was also significantly lower (Fig. 7b). Histopathological examination of the colon revealed a reduced degree of colonic damage in T-bet^ΔNCR+ILC^ mice (Fig. 7c, d). There was no difference in colitis between WT mice or control mice containing only the *Ncr1*-iCre transgene, or the floxed T-bet allele (T*-bet*^*fl/fl*^*)* (Supplemental Fig. 10). Strikingly, marked eosinophilic infiltration was observed in T-bet^ΔNCR+ILC^ mice (Fig. 7d, e), whereas the presence of neutrophils in the cLP in WT and T-bet^ΔNCR+ILC^ mice was very similar (Fig. 7f). Eosinophil activation and accumulation in tissues during inflammation has been attributed to resident ILC2s through IL-5 and IL-13 expression.^43^ Thus, we evaluated IL-13 production in ex vivo colonic organ cultures, finding increased levels of this cytokine in T-bet^ΔNCR+ILC^ mice (Fig. 7g). In addition, qPCR analysis of cytokine transcripts in colonic tissue revealed high expression of IL-13, IL-5 and IL-4 in colitic T-bet^ΔNCR+ILC^ mice (Fig. 7h) while no differences were observed in IL-17A or IFNγ expression or production (Fig. 7g, h). Furthermore, cLP ILCs from T-bet^ΔNCR+ILC^ DSS-colitis mice produced higher IL-13 and IL-5 after stimulation than ILCs from DSS-WT mice (Fig. 7i), whereas IL-17A and IFNγ production by ILCs were equivalent in both groups (Fig. 7j). Therefore, T-bet deletion in ILCs may lead to the development of a type 2 mucosal immune response after an inflammatory insult with the potency of attenuating tissue damage.

## Discussion

Here, we report a previously unappreciated role for T-bet in the homoeostasis and function of ILC2s. We have demonstrated that T-bet deficiency in ILCs favours ILC2 responses, conferring protection against intestinal parasites and modulating the immune response during colitis. In this context, T-bet expression in ILCs in the absence of an adaptive immune system works to dampen pro-inflammatory responses, as TRUC mice have been found to develop colitis driven by aberrant numbers of IL-17-producing ILCs.^5^ By contrast, we now report that in a more physiologically relevant setting, in the presence of an otherwise intact immune system, T-bet deficiency in ILCs results in the development of protective mucosal immune responses.

We have established a new model of selective deletion of T-bet in ILCs, the T-bet^ΔNCR+ILC^ mouse. The effects of T-bet in other immune cell types can interfere with or disguise the phenotypes attributed to ILCs. We have shown that as opposed to what observed in animals with a germline deletion of T-bet, NCR^+^ ILCs can be found in the T-bet^ΔNCR+ILC^ model, and the function and development of these and other NKp46-expressing cells remains largely unaffected. By developing this model, we have also provided further insights into the role of T-bet in NCR^+^ ILC development. In contrast to what has been previously reported for *T-bet*^*−/−*^mice,^2,4^ when T-bet deletion is under the control of *Ncr1* expression, NCR^+^ ILCs can be found in the intestinal mucosa in the same numbers as in T-bet-sufficient animals, and their ability to produce IFNγ is unaffected.

In addition, by using the T-bet^ΔNCR+ILC^ model we have confirmed a role for T-bet in controlling ILC2 responses, although these cells express no detectable amounts of T-bet. IFNγ has been reported to limit type 2 cytokine expression in lung ILC2s, however,^44–46^ ILC2 expansion in the absence of T-bet is independent of IFNγ as the number of ILC2s in IFNγ-deficient mice was not altered. One of the potential mechanisms of T-bet mediated control of the intestinal ILC pool, is in the regulation of IL-7 signalling. Similar to our previous findings in CD4^+^ T-cells where we demonstrated that T-bet binds to the IL-7Rα promoter,^5^ T-bet acts as a transcriptional repressor of the *Il7ra* gene in ILCs, and increased IL-7Rα expression in ILCs from a T-bet-deficient background leads to increased activation of STAT-5 upon IL-7 stimulation. Importantly, we have found that the enhanced ILC expression of IL-7Rα in the absence of T-bet occurs in the context of normal GATA-3 levels, providing further evidence that T-bet is acting as a direct transcriptional repressor of IL-7Rα, rather than indirectly via a change in GATA-3 expression. Increased IL-7 responsiveness in the absence of retinoic acid signalling has been related to higher ILC2 proliferation and accumulation.^21^ Interestingly, we have demonstrated that RARα and T-bet interact to control T-cell plasticity and lineage stability, providing a potential unifying mechanism for these observations.^47^ STAT-5 activation may stabilise GATA-3 expression in ILCs, which would drive ILC2 differentiation and account for the higher numbers of ILC2s observed in T-bet-deficient animals. Genome-wide analysis has revealed how T-bet and GATA-3 binding sites are related to each other.^48,49^ In Th1 cells T-bet sequesters GATA-3 away from Th2 genes preventing their activation, and loss of T-bet induces default commitment to Th2 and Th17 lineages.^50^ Similarly, T-bet could also interact with GATA-3 in ILCs, and it is therefore possible that T-bet loss in ILCs induces default commitment to ILC2s. In fact, transitioning regulatory elements in ILC precursors have been recently reported to be enriched with motifs for both T-box and GATA-3 families, suggesting the existence of a prior stage in which the level of these two transcription factors could determine a particular ILC fate.^51^ Therefore, T-bet deficiency in ILCs could potentially allow for the activation of GATA-3 related genes mediating the expansion of group 2 ILCs.

The transcriptional control of ILCs represents an important mechanism by which T-bet regulates immune responses that is likely to be relevant for understanding the pathogenesis of different inflammatory conditions. In the intestinal mucosa, ILCs are potent innate immune effector cells that promote resistance to pathogens and maintain homoeostasis.^52^ Group 2 ILCs respond to epithelial-derived cytokines such as IL-25 and IL-33 and promote immunity to intestinal parasites in mice.^17,18,38,39^ We have shown that increased ILC2 numbers in T-bet-deficient mice promote early worm expulsion upon *T. spiralis* infection. Moreover, challenging these mice with DSS resulted in the development of a milder intestinal inflammatory process. Instead of IFNγ- and IL-17-mediated colitis, these mice developed a type 2 intestinal immune response that prevented the deleterious effects of an aberrant activation of the immune system. These results suggest that T-bet expression in ILCs can regulate both pro- and anti-inflammatory processes in the intestinal mucosa. Thus, tuning the levels of T-bet expression in ILCs could potentially represent a tool to optimise immune responses, allowing a selective regulation of protective vs. pathological ILC responses, which could be relevant for the treatment of IBD and other ILC-mediated conditions.

Our results here shed new light on the role that T-bet plays in ILC homoeostasis and function in health and disease. T-bet has been proven essential for ILC1 and ILC3 biology. We have now demonstrated that T-bet also modulates ILC2 responses in the intestinal lamina propria. Therefore, we propose T-bet as a critical mediator of ILC responses, establishing a mechanism for its role as a key regulator of mucosal immunity.

## Methods

### Animals

BALB/c and C57BL/6 WT (Charles River) and *T-bet*^*−/−*^ and *RAG2*^*−/−*^ (Jackson labs) mice were sourced commercially. *T-bet*^*fl/fl*^ mice were previously generated by our group.^19^ *Ncr1*-iCre^Tg^ mice were a gift from Veronica Sexl, University of Veterinary Medicine Vienna^35^ and tissues from *Ifng*^-/-^ mice were provided by Professor Anne O’Garra, The Francis Crick Institute, UK. TRnUC and T-bet^ΔNCR+ILC^ mouse strains were bred locally by breeding BALB/c *RAG2*^*−/−*^ and *T-bet*^*−/−*^ mice, and *Ncr1*-iCre^Tg^ and *T-bet*^*fl/fl*^ mice respectively. *T-bet*^*fl/fl*^, *Ncr1*-iCre^Tg^ littermates and C57BL/6 mice were all used as controls for experiments involving T-bet^ΔNCR+ILC^ mice; no differences were observed between *T-bet*^*fl/fl*^ and *Ncr1*-iCre^Tg^ mice when compared to C57BL/6 animals in any of the experiments. Male mice of 6-12 weeks of age were used. All mice were housed in specific pathogen–free facilities at King’s College London Biological Services Unit, University of Manchester Biological Services Facility or at Charles River Laboratories.

### Isolation of cells

cLP cells were isolated as previously described^53^ using a digestion media composed of HBSS without Mg^2+^ or Ca^2+^ (Invitrogen), 2% of fetal calf serum (FCS Gold, PAA Laboratories), and 0.5 mg/ml collagenase D, 10 μg/ml DNase I and 1.5 mg/ml dispase II (all Roche). The cLP lymphocyte-enriched population was harvested from a 40–80% Percoll (GE Healthcare) gradient. Splenic and MLN cells were isolated by mechanical dissociation through 70-μm filters into a single-cell suspension.

### Flow cytometry

Flow cytometry was performed as previously described.^5^ GATA-3 staining was performed using the Transcription factor buffer set and pSTAT-5, 3 and 4 were measured using BD Phosflow kit (BD Biosciences). In phosphoflow experiments cells were stimulated with IL-7 (50 ng/ml) or IL-2 (50 ng/ml) in cytokine-free ex vivo 20 medium (Lonza) at 37 °C for 30 or 60 min. Antibodies were from eBioscience unless otherwise stated. A lineage cocktail was used including antibodies against CD3, CD45R, CD11b, TER-119 and Ly-6G, and additional CD5, CD19 and FcεRI in some experiments. For a complete list of the antibodies used see supporting documents. Samples were acquired using an LSRFortessa™ cell analyser (Becton Dickinson, USA) and data were analysed using FlowJo software (Tree Star, USA).

### Cell sorting

Single-cell suspensions were stained with fluorescently labelled antibodies as described and analysed and sorted (purity > 98%) using a BD FACSAria III cell sorter (BD Biosciences). For ILCs, antibodies against CD45, lineage markers and IL-7Rα were used to separate CD45^+^Lin^−^IL-7Rα^+^ cells. ILC2s were sorted as ICOS^+^CD25^+^cKit^+^CCR6^−^ ILCs (spleen) and KLRG1^+^ICOS^+^CCR6^−^ ILCs (cLP).

### In vitro studies

For ILC2s cytokine production assays in vitro, unfractionated lymphocytes depleted of CD4^+^ T-cells using MACS MicroBead Technology (Miltenyi Biotec) or FACS-sorted ILC2s from the spleen and cLP of *T-bet*^*−/−*^ and WT mice were cultured at a concentration of 5 × 10^6^ lymphocytes/ml or 400.000 ILC2s/ml in complete RPMI-1640 medium (PAA Laboratories) supplemented with 10% FCS, 2mM l-glutamine and nonessential amino acids (Sigma-Aldrich), 10 mM HEPES (Fisher Scientific), and 1 mM sodium pyruvate, 50 μM 2-mercaptoethanol, 100 IU/ml penicillin and 100 μg/ml streptomycin (Invitrogen). IL-25 and/or IL-33 (50 ng/ml), or PMA (50 ng/ml) and ionomycin (1 μg/ml) were added to the cultures. After 24 h of culture at 37 °C, supernatants were collected and stored at –80 °C until used for cytokine measurement by ELISA.

For in vitro TAT-Cre recombinase deletion of T-bet, 1 × 10^6^ cells/ml FACS-sorted ILCs from *T-bet*^*fl/fl*^ mice were cultured in serum-free complete RPMI-1640 medium. TAT-Cre recombinase (100 μg/ml) (EMD Millipore) was added to the cultures for 1 h, and then washed with media supplemented with 20% FCS. The cells were kept in culture with IL-2 (100UI/ml) and IL-7 (10 ng/ml) for a week. Intracellular cytokine, surface markers and transcription factor expression were analysed by flow cytometry. For IL-13 determination cells were previously stimulated with IL-25 and IL-33 (50 ng/ml) and after 24 h of culture at 37 °C, supernatants were collected and stored at –80 °C until used for cytokine measurement by ELISA. In separate experiments, cells were isolated after 4 h and analysed by flow cytometry.

### *Trichinella spiralis* infection

The maintenance, infection and recovery of *T. spiralis* was carried out as previously described.^54^ WT, *T-bet*^*−/−*^and T-bet^ΔNCR+ILC^ mice were infected with 300 *T. spiralis* muscle larvae and were sacrificed 8 or 14 days after the infection. At necropsy representative whole gut specimens (0.5 cm length) were taken from the SI (9 cm from the pylorus) for histology studies and RNA extraction. Adult worms were recovered from the SI and counted as previously described.^55^ Spleen and MLN were harvested for flow cytometry.

### DSS-induced colitis

Colitis was induced by adding 3% DSS (36-50 KDa, MP Biomedicals, Ontario, USA) to the drinking water for 5 days. Non-colitic mice were administered sterile drinking water. Mice were sacrificed 10 days after the beginning of the experiment. An average daily DAI score was calculated according to the criteria proposed previously.^56^ At necropsy, representative whole gut specimens (0.5 cm length) were taken from the distal inflamed region of the colon for histology studies and RNA extraction. In all, 3 mm punch biopsies (Miltex) were obtained for colon explant cultures, performed as described previously.^5^ The remaining colonic tissue was used for LP cell isolation and flow cytometry.

### Histology

Colon and SI cross-sections were fixed in 10% paraformaldehyde and embedded in paraffin blocks. Full-thickness sections of 5 μm were stained with haematoxylin and eosin (H&E). SI muscle thickness, villus length and crypt length in *T. spiralis* experiments and colonic microscopic damage in DSS-colitis experiments (criteria described in Supplemental table) were evaluated by pathologist observers (AG and TM, respectively) who were blinded to the experimental groups.

### Cytokine determination

Intracellular cytokine expression was measured by flow cytometry after cells were stimulated with PMA (50 ng/ml) and ionomycin (1 μg/ml) for 4 h. IL-13 concentration in culture supernatants was measured by ELISA (eBioscience) and IFNγ and IL-17A concentration was measured using a BD Cytometric Bead Array Mouse Th1/Th2/Th17 Cytokine kit (BD biosciences).

### Real-time PCR

Snap frozen colon segments were homogenised using a Tissue lyzer II with a Stainless-Steel Bead (5 mm) and RNA was extracted using a RNeasy^®^ Mini Kit (all Qiagen). cDNA was generated with a cDNA synthesis kit (Bioline). mRNA transcripts were quantified by quantitative PCR using TaqMan gene expression assays for IL-13 (Mm0043204_m1), IL-5 (Mm00439646_m1), IL-4 (Mm00445259_m1), IFNγ (Mm01168134_m1) and IL-17A (Mn00439619_m1) (Applied Biosystems, Warrington, UK). Gene expression was normalised to the expression of β-actin (4352341E) to generate ΔCT values and relative abundance was quantified using the 2^−ΔCT^ method.

### Western blotting

T-bet expression analysis by western blot was performed as described elsewhere.^57^ Anti-T-bet antibody (eBio4B10, eBioscience) was used at a dilution of 1/1000. A primary antibody against β-actin (1/1000) (13E5, Cell Signalling Technology, Danvers, USA) was used as loading control. Peroxidase-conjugated anti-mouse IgG (1/5000) (GE Healthcare UK Limited) was used as secondary antibody. All antibody incubations were performed at 4 °C overnight.

### Statistics

Results are expressed as mean ± SEM. Non−parametric data were analysed using a Mann−Whitney *U*-test or two-way analysis of variance (ANOVA), parametric data was analysed using a two-tailed *t*-test, as appropriate, using GraphPad Prism 5.0 (GraphPad Inc., USA). ns: non^−^significant; *p < 0.05; **p < 0.01; ***p < 0.001; ****p < 0.0001.

## Electronic supplementary material

Supplementary data

Supplementary Figures
