# Supplementary material for: T-bet controls intestinal mucosa immune responses via repression of type 2 innate lymphoid cell function
Source: Mucosal Immunol. 2018 Oct 24;12(1):51–63. doi: 10.1038/s41385-018-0092-6 (PMC6548562; doi:10.1038/s41385-018-0092-6)
Supplement: Supplementary file 1 — Supplementary data [file 41385_2018_92_MOESM1_ESM.docx]

SUPPLEMENTARY INFORMATION

**Supplementary Materials and Methods**

**Flow cytometry**

Antibodies were from eBioscience unless otherwise stated: α-mouse CD45 (30-F11, Invitrogen), α-mouse CD127 (A7R34), α-mouse NKp46 (29A1.4), α-mouse CD25 (PC61.5), α-mouse c-Kit (2B8), α-mouse ICOS (C398.4A), α-mouse KLRG1 (2F1), α-mouse CCR6 (29-2L17, Biolegend), α-mouse CD90.2 (53-2.1), α-mouse NK1.1 (PK136), α-mouse CD27 (LG.7F9), α-mouse CD11b (M1/70), α-mouse F4/80 (BM8), α-mouse Ly6C (HK1.4), α-mouse Siglec F (REA798), α-mouse CCR3 (J073E5), α-mouse IL-5 (TRFK5), α-mouse IL-13 (eBio13A), α-mouse IFNγ (XMG1.2), α-mouse IL-17A (eBio17B7), α-human/α-mouse T-bet (eBio4B10), α-mouse RORγt (B2D), α-human/α-mouse GATA-3 (TWAJ), α-mouse pSTAT-5 (pY694, BD Biosciences), α-mouse pSTAT-4 (PY693), α-mouse pSTAT-3 (LUVNKLA) and α-mouse Ki67 (SolA15). A lineage cocktail was used including the following antibodies: α -mouse CD3 (17A2), α-mouse CD45R (RA3-6B2), α -mouse CD11b (M1/70), α -mouse TER-119 (TER-119), α -mouse Ly-6G (RB6-8C5). For additional experiments α-mouse CD5 (53-7.3), α-mouse CD19 (eBio 1D3) and α-mouse FcεRI (MAP-1) were also included in the lineage cocktail.

**Naïve T cells skewing**

Naïve T cells (CD4+ CD25- cells) were selected from single-cell suspensions from the spleen using mouse CD4 and CD25 MicroBeads (Miltenyi Biotec) following the manufacturer’s instructions. They were cultured at a concentration of 1x106 cells/ml in 48 well/plates previously coated with αCD3 and αCD28 antibodies, in Th1 or Th2 skewing media. For Th1 differentiation, complete media was supplemented with αIL-4 (5μg/ml), IL-12 (20ng/ml) and IL-2 (20ng/ml) and for Th2 differentiation it contained αIFNγ (20μg/ml), IL-4 (20ng/ml) and IL-2 (20ng/ml) (BioLegend). Skewing cytokines were added to RPMI-1640 medium (PAA Laboratories) supplemented with 10% FCS (PAA Laboratories), 50 μM 2-mercaptoethanol (Invitrogen), 2mM L-glutamine (Sigma-Aldrich), 1 mM sodium pyruvate (Invitrogen), 10 mM HEPES (Fisher Scientific), nonessential amino acids (Sigma-Aldrich), 100 IU/ml penicillin, and 100 μg/ml streptomycin (Invitrogen). Cultures were kept for 5 days and new media and skewing cytokines were added every two days.

**NK cells *in vitro* stimulation**

NK cells were positively selected from single-cell suspensions from the spleen and mLN using mouse CD49b MicroBeads (Miltenyi Biotec) and following the manufacturer’s instructions. They were cultured at a concentration of 1x106 cells/ml in complete RPMI-1640 medium as described above. IL-12 and IL-18 were added to the cultures at a concentration of 10ng/ml. After 5 or 24 hours of culture at 37°C, cells were collected and IFN-γ production was assessed by intracellular staining and flow cytometry using an α-mouse IFN-γ (XMG1.2) antibody (eBioscience).

**Supplementary Table**

Scoring criteria of full-thickness distal colon sections from DSS-colitis mice.

| **Mucosal epithelium and lamina propria** |  |
| --- | --- |
| Ulceration: none (0); mild surface (0-25%) (1); moderate (25-50%) (2); severe (50-75%) (3); extensive-full thickness (more 75%) (4).  Polymorphonuclear cell infiltrate  Mononuclear cell infiltrate and fibrosis  Edema and dilation of lacteals |  |
| **Crypts** |  |
| Hyperplasia/disrupted architecture  Dilations  Goblet cell depletion: none (0); <10% (1); 15-25%) (2); 25-50%) (3); more 50%) (4). |  |
| **Submucosa** |  |
| Polymorphonuclear cell infiltrate  Mononuclear cell infiltrate  Edema |  |
| **Muscular layer** | |
| Polymorphonuclear cell infiltrate  Mononuclear cell infiltrate  Edema  Infiltration in the serosa | |

Scoring scale: 0, none; 1 slight; 2, mild; 3, moderate; 4, severe.

Maximum score: 56.
