# Supplementary material for: T-bet controls intestinal mucosa immune responses via repression of type 2 innate lymphoid cell function
Source: Mucosal Immunol. 2018 Oct 24;12(1):51–63. doi: 10.1038/s41385-018-0092-6 (PMC6548562; doi:10.1038/s41385-018-0092-6)
Supplement: Supplementary file 2 — Supplementary Figures [file 41385_2018_92_MOESM2_ESM.pptx]

## Slide 1
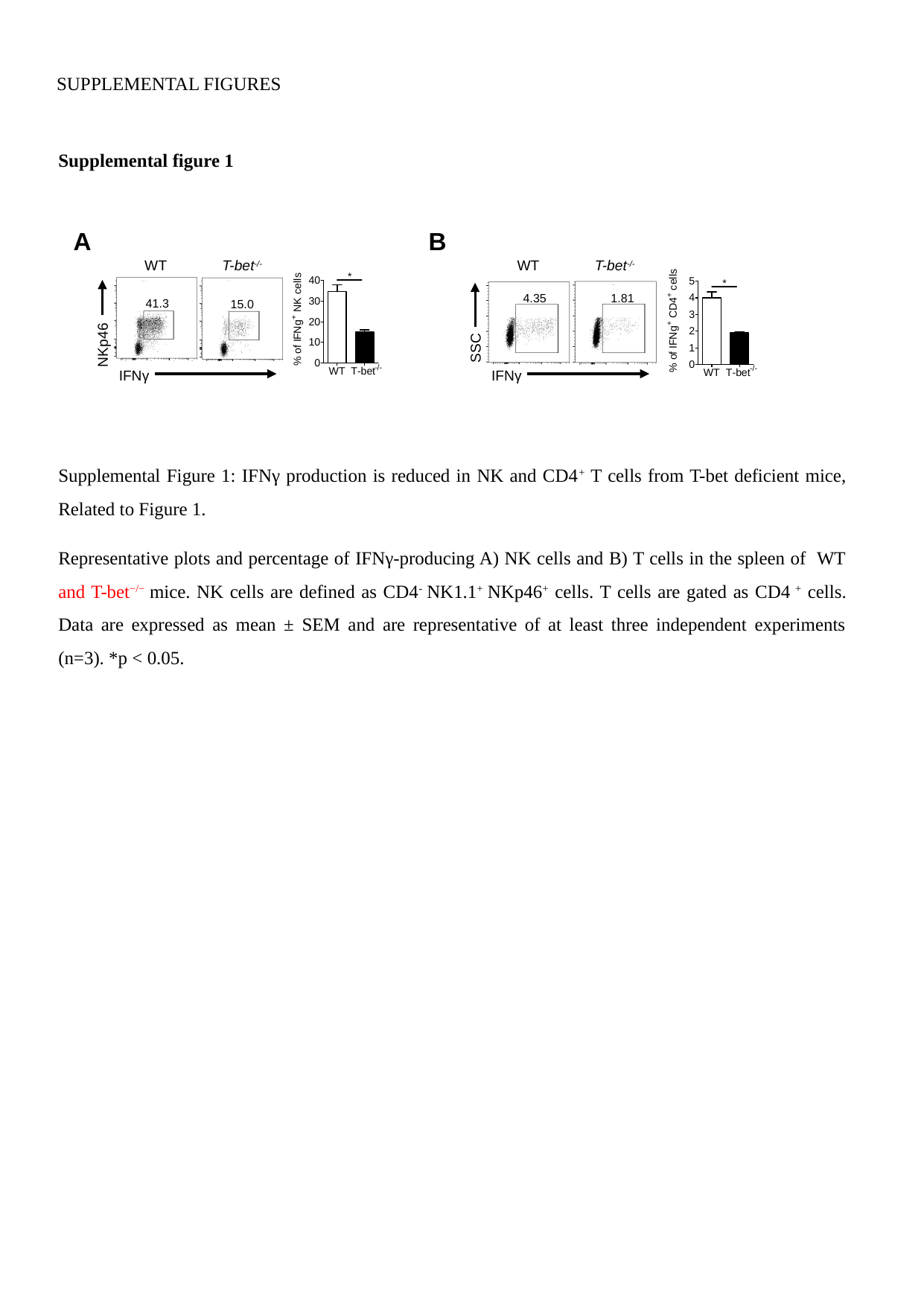

SUPPLEMENTAL FIGURES
Supplemental figure 1
A
B
WT
T-bet-/-
41.3
15.0
NKp46
IFNγ
WT
T-bet-/-
4.35
1.81
SSC
IFNγ
Supplemental Figure 1: IFNγ production is reduced in NK and CD4+ T cells from T-bet deficient mice, Related to Figure 1.
Representative plots and percentage of IFNγ-producing A) NK cells and B) T cells in the spleen of WT and T-bet−/− mice. NK cells are defined as CD4- NK1.1+ NKp46+ cells. T cells are gated as CD4 + cells. Data are expressed as mean ± SEM and are representative of at least three independent experiments (n=3). *p < 0.05.

## Slide 2
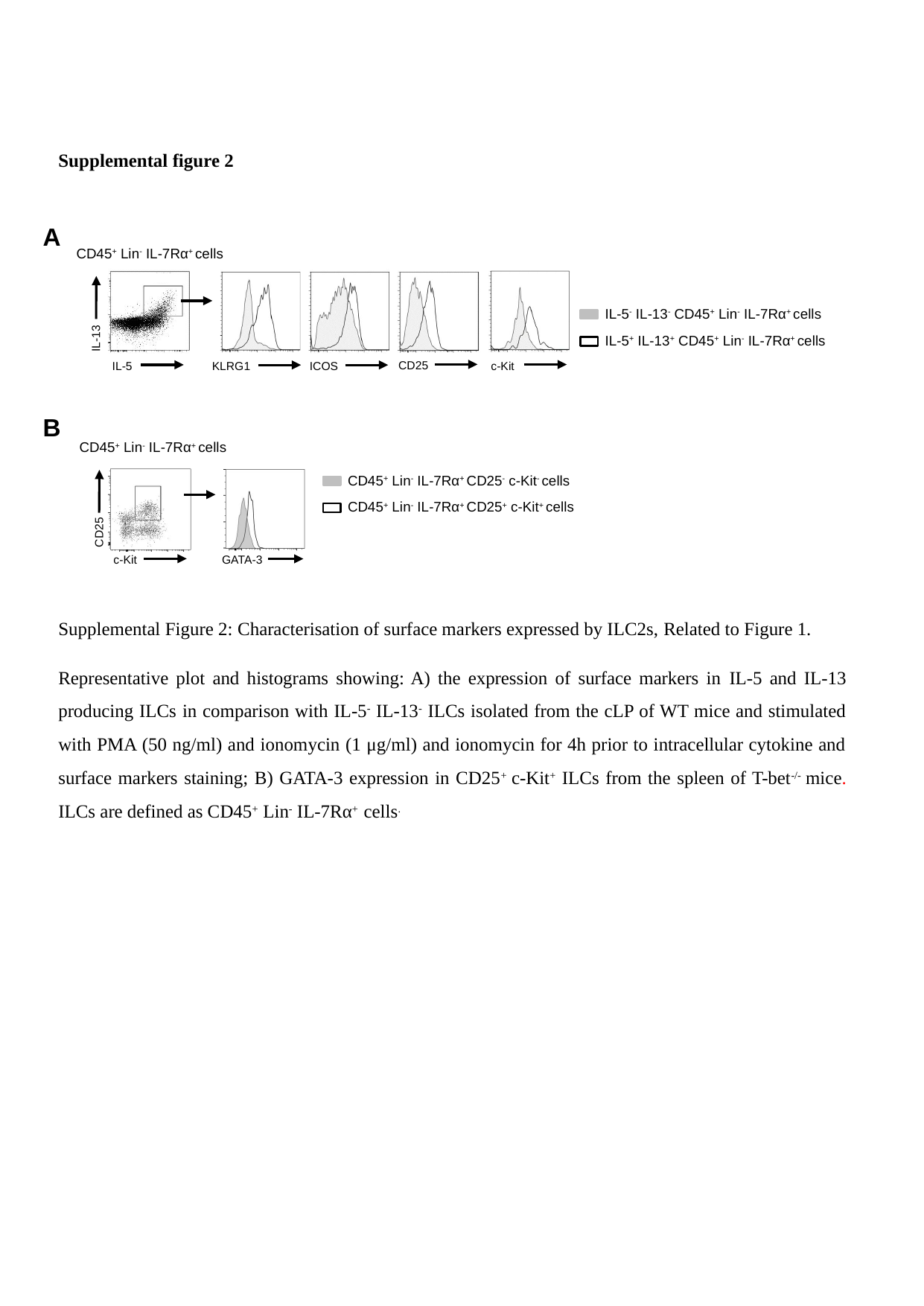

Supplemental figure 2
A
CD45+ Lin- IL-7Rα+ cells
IL-13
CD25
c-Kit
IL-5
ICOS
KLRG1
IL-5- IL-13- CD45+ Lin- IL-7Rα+ cells
IL-5+ IL-13+ CD45+ Lin- IL-7Rα+ cells
B
CD45+ Lin- IL-7Rα+ cells
CD45+ Lin- IL-7Rα+ CD25- c-Kit- cells
CD45+ Lin- IL-7Rα+ CD25+ c-Kit+ cells
CD25
GATA-3
c-Kit
Supplemental Figure 2: Characterisation of surface markers expressed by ILC2s, Related to Figure 1.
Representative plot and histograms showing: A) the expression of surface markers in IL-5 and IL-13 producing ILCs in comparison with IL-5- IL-13- ILCs isolated from the cLP of WT mice and stimulated with PMA (50 ng/ml) and ionomycin (1 μg/ml) and ionomycin for 4h prior to intracellular cytokine and surface markers staining; B) GATA-3 expression in CD25+ c-Kit+ ILCs from the spleen of T-bet-/- mice. ILCs are defined as CD45+ Lin- IL-7Rα+ cells.

## Slide 3
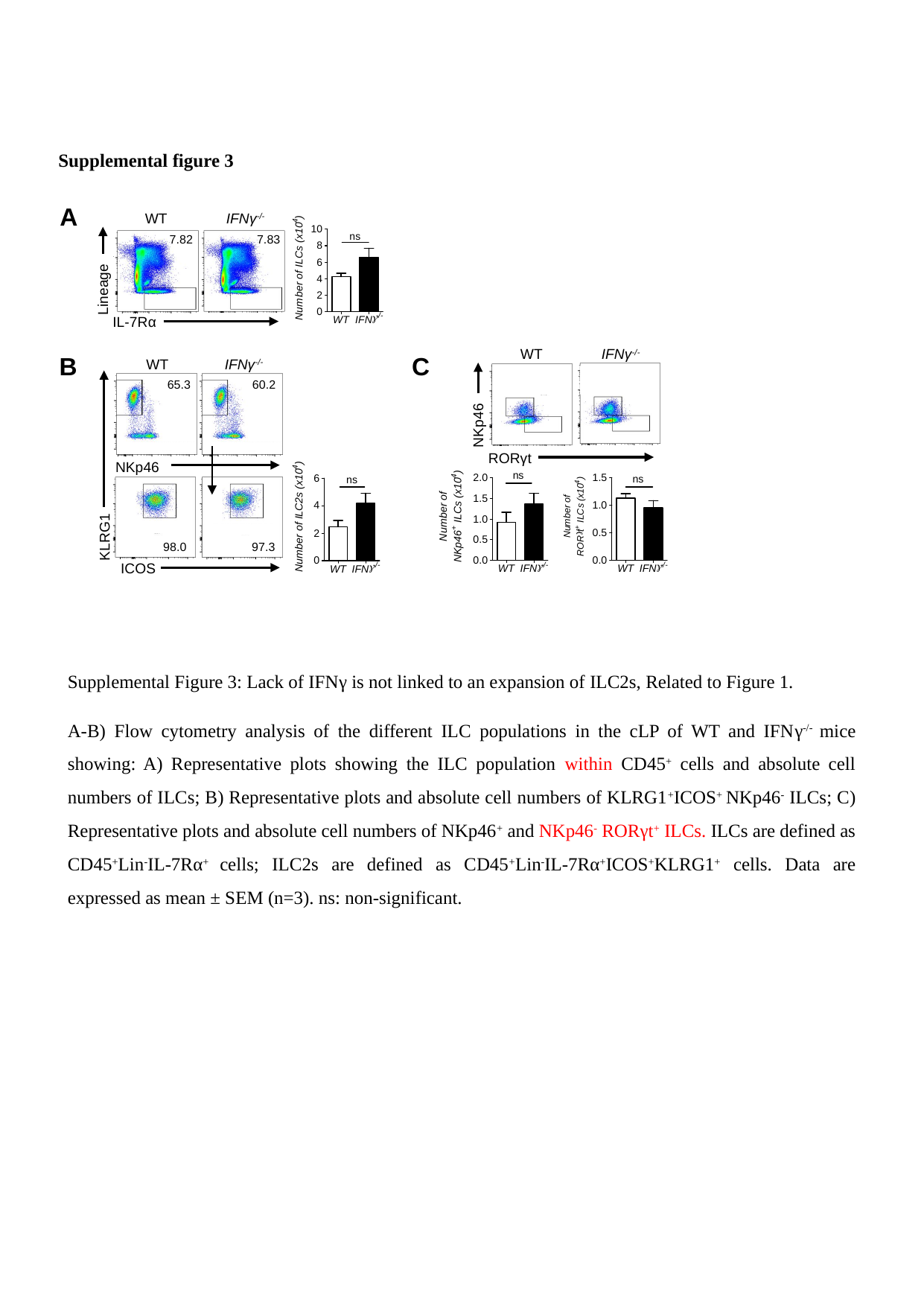

Supplemental figure 3
A
WT
IFNγ-/-
7.82
7.83
IL-7Rα
Lineage
WT
IFNγ-/-
NKp46
RORγt
B
C
WT
IFNγ-/-
65.3
60.2
NKp46
KLRG1
98.0
97.3
ICOS
Supplemental Figure 3: Lack of IFNγ is not linked to an expansion of ILC2s, Related to Figure 1.
A-B) Flow cytometry analysis of the different ILC populations in the cLP of WT and IFNγ-/- mice showing: A) Representative plots showing the ILC population within CD45+ cells and absolute cell numbers of ILCs; B) Representative plots and absolute cell numbers of KLRG1+ICOS+ NKp46- ILCs; C) Representative plots and absolute cell numbers of NKp46+ and NKp46- RORγt+ ILCs. ILCs are defined as CD45+Lin-IL-7Rα+ cells; ILC2s are defined as CD45+Lin-IL-7Rα+ICOS+KLRG1+ cells. Data are expressed as mean ± SEM (n=3). ns: non-significant.

## Slide 4
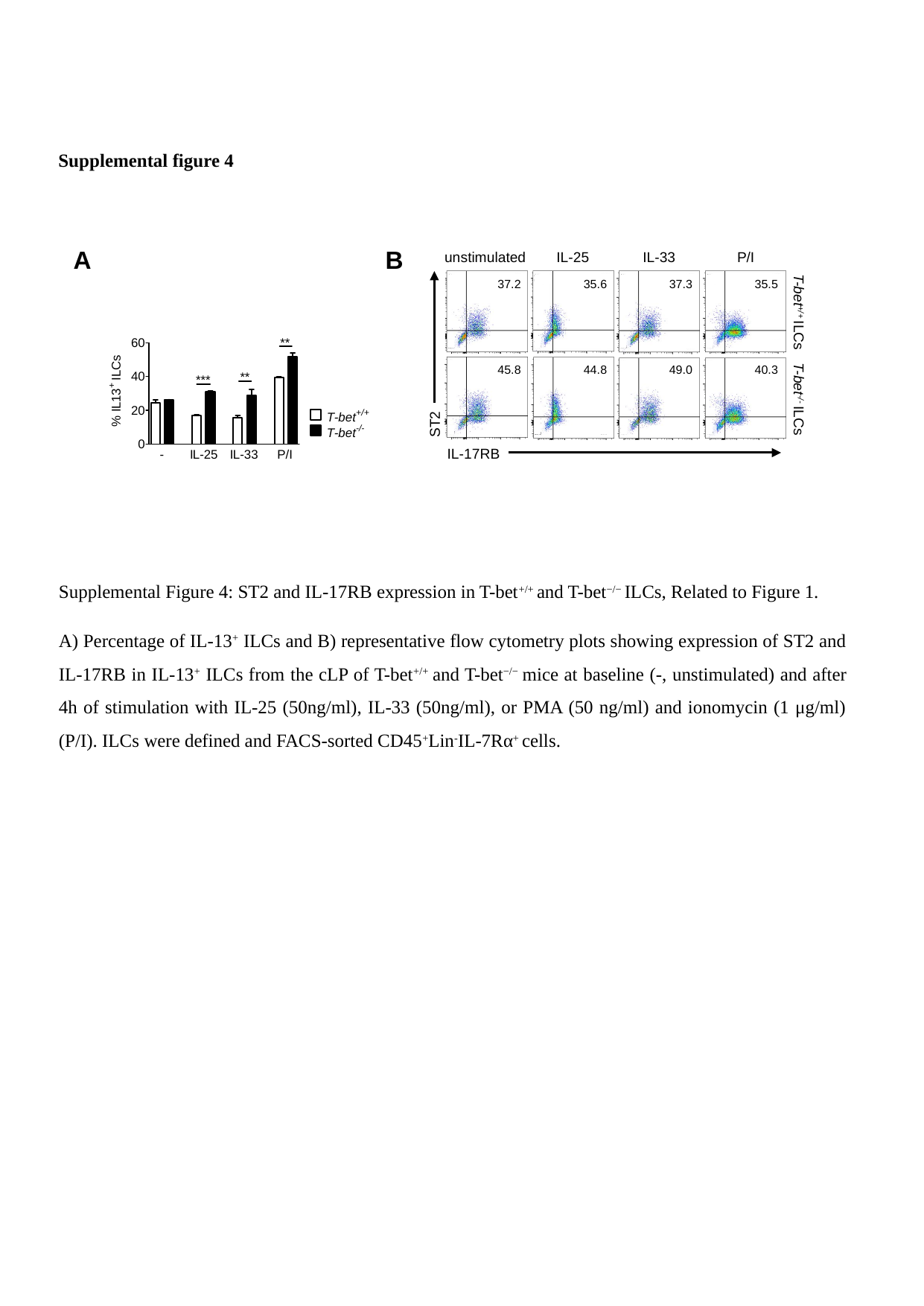

Supplemental figure 4
A
B
unstimulated
IL-25
IL-33
P/I
37.2
35.6
37.3
35.5
T-bet+/+ ILCs
45.8
44.8
49.0
40.3
T-bet-/- ILCs
ST2
IL-17RB
Supplemental Figure 4: ST2 and IL-17RB expression in T-bet+/+ and T-bet−/− ILCs, Related to Figure 1.
A) Percentage of IL-13+ ILCs and B) representative flow cytometry plots showing expression of ST2 and IL-17RB in IL-13+ ILCs from the cLP of T-bet+/+ and T-bet−/− mice at baseline (-, unstimulated) and after 4h of stimulation with IL-25 (50ng/ml), IL-33 (50ng/ml), or PMA (50 ng/ml) and ionomycin (1 μg/ml) (P/I). ILCs were defined and FACS-sorted CD45+Lin-IL-7Rα+ cells.

## Slide 5
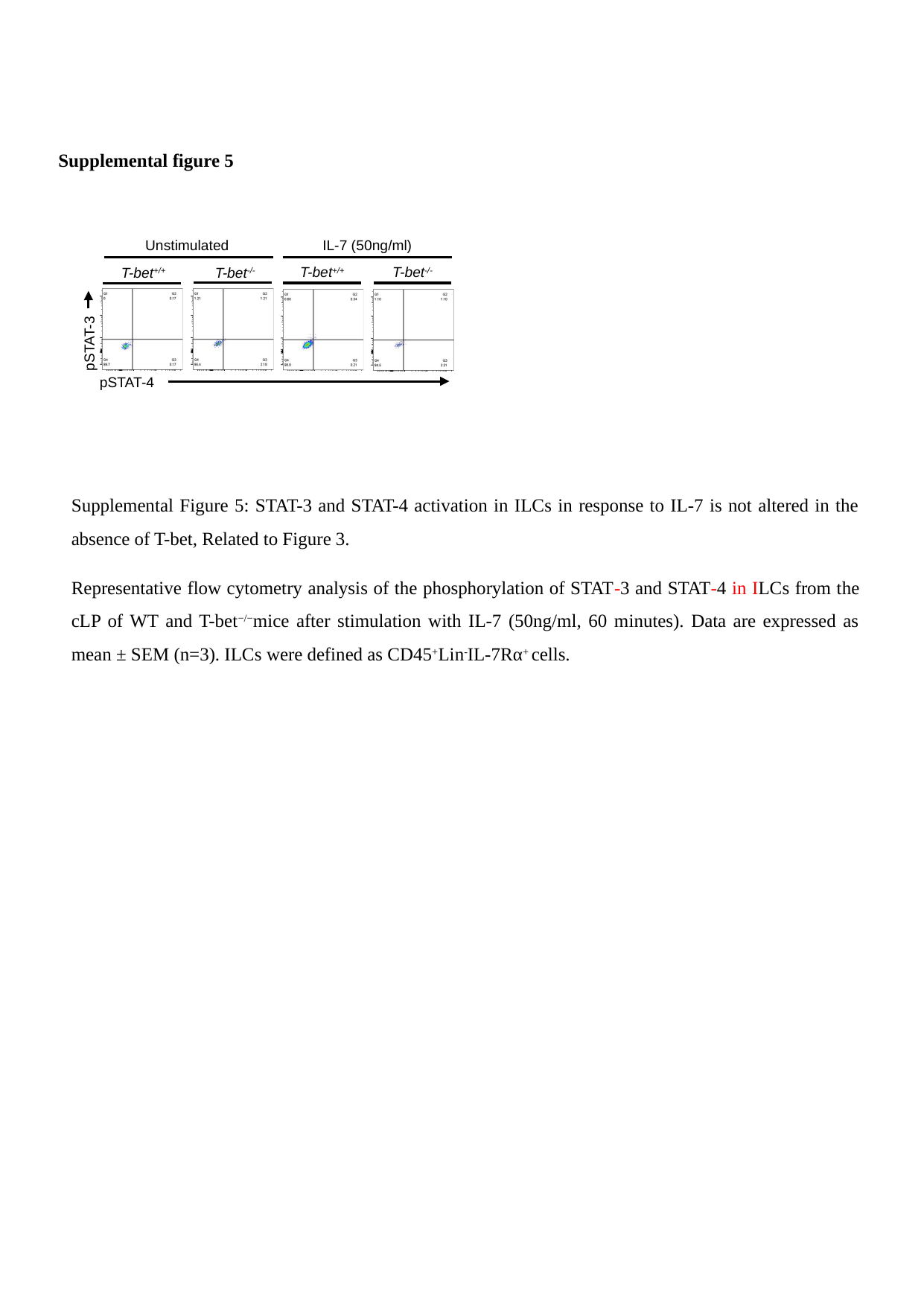

Supplemental figure 5
Unstimulated
IL-7 (50ng/ml)
T-bet+/+
T-bet-/-
T-bet+/+
T-bet-/-
pSTAT-3
pSTAT-4
Supplemental Figure 5: STAT-3 and STAT-4 activation in ILCs in response to IL-7 is not altered in the absence of T-bet, Related to Figure 3.
Representative flow cytometry analysis of the phosphorylation of STAT-3 and STAT-4 in ILCs from the cLP of WT and T-bet−/−mice after stimulation with IL-7 (50ng/ml, 60 minutes). Data are expressed as mean ± SEM (n=3). ILCs were defined as CD45+Lin-IL-7Rα+ cells.

## Slide 6
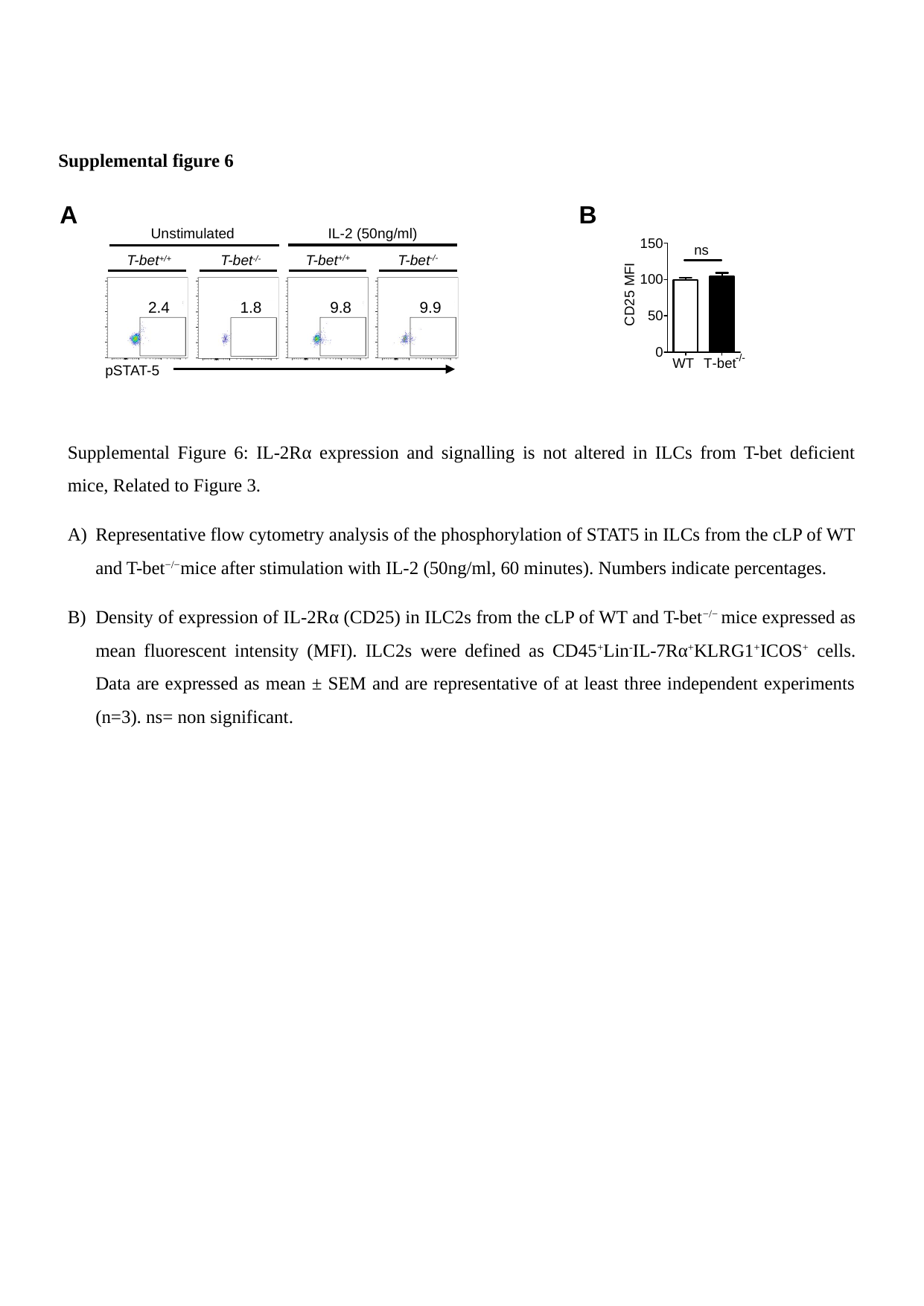

Supplemental figure 6
A
B
Unstimulated
IL-2 (50ng/ml)
T-bet+/+
T-bet-/-
T-bet+/+
T-bet-/-
2.4
1.8
9.8
9.9
pSTAT-5
Supplemental Figure 6: IL-2Rα expression and signalling is not altered in ILCs from T-bet deficient mice, Related to Figure 3.
Representative flow cytometry analysis of the phosphorylation of STAT5 in ILCs from the cLP of WT and T-bet−/−mice after stimulation with IL-2 (50ng/ml, 60 minutes). Numbers indicate percentages.
Density of expression of IL-2Rα (CD25) in ILC2s from the cLP of WT and T-bet−/− mice expressed as mean fluorescent intensity (MFI). ILC2s were defined as CD45+Lin-IL-7Rα+KLRG1+ICOS+ cells. Data are expressed as mean ± SEM and are representative of at least three independent experiments (n=3). ns= non significant.

## Slide 7
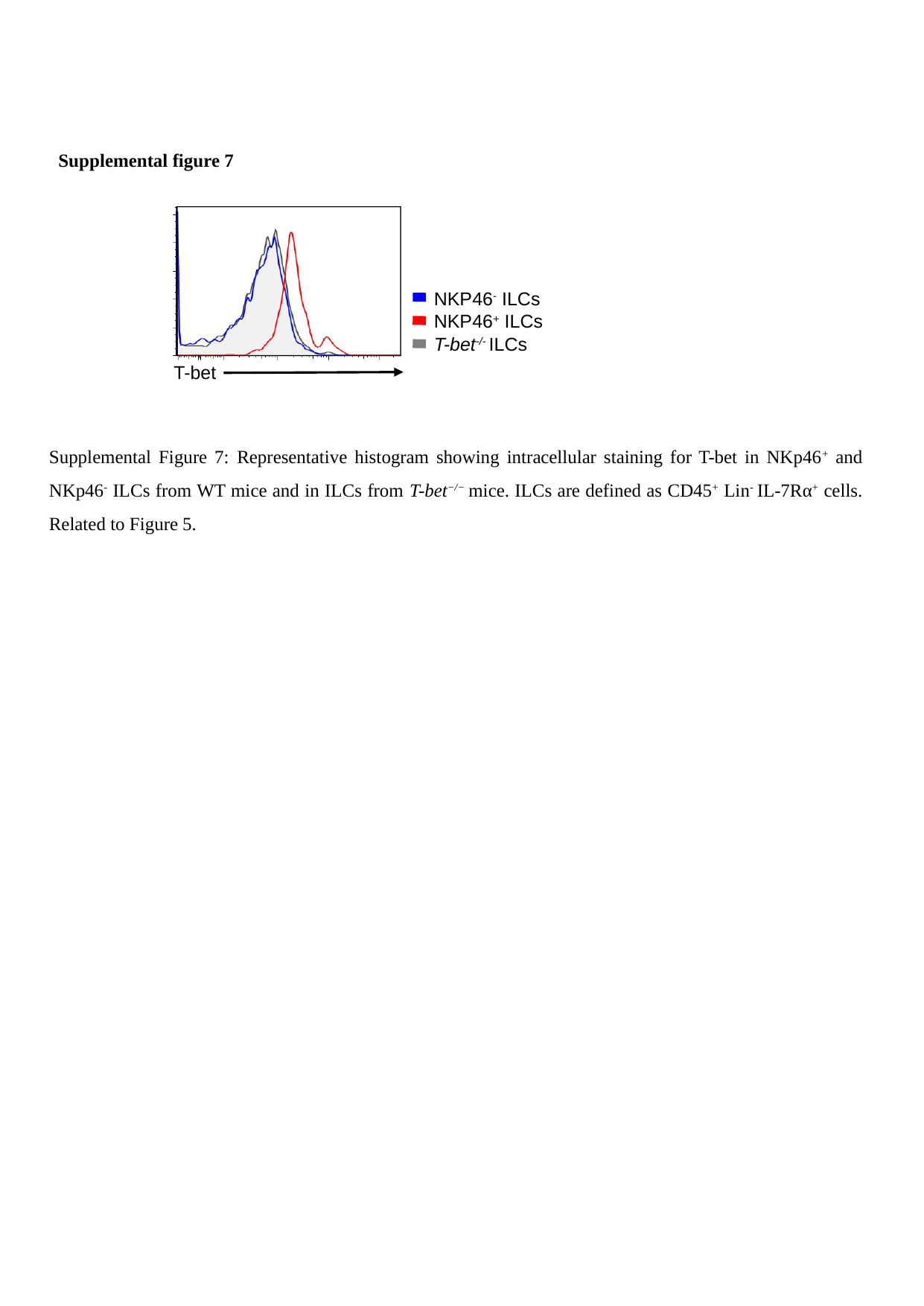

Supplemental figure 7
NKP46- ILCs
NKP46+ ILCs
T-bet-/- ILCs
T-bet
Supplemental Figure 7: Representative histogram showing intracellular staining for T-bet in NKp46+ and NKp46- ILCs from WT mice and in ILCs from T-bet−/− mice. ILCs are defined as CD45+ Lin- IL-7Rα+ cells. Related to Figure 5.

## Slide 8
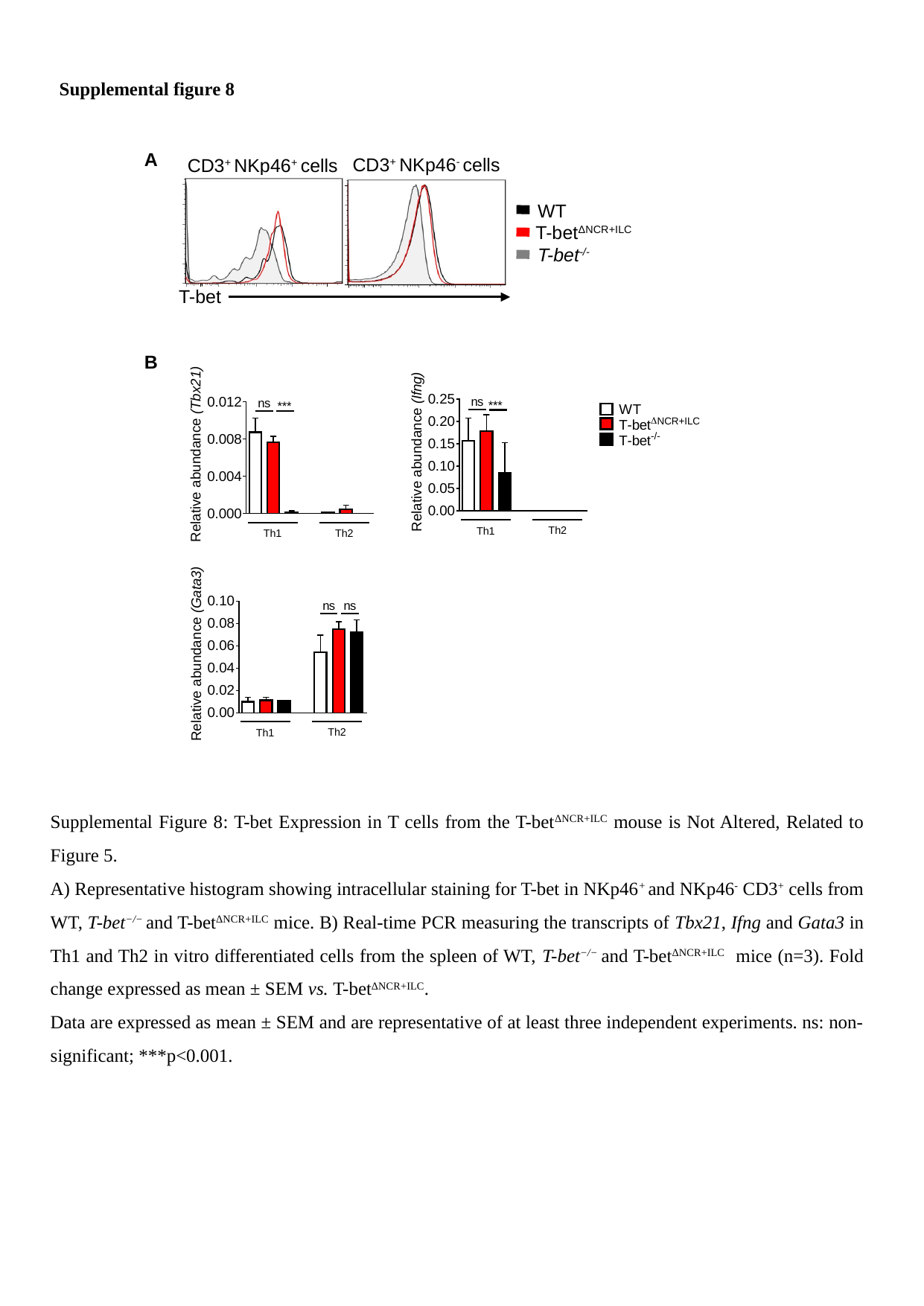

Supplemental figure 8
A
CD3+ NKp46- cells
CD3+ NKp46+ cells
T-bet
WT
T-betΔNCR+ILC
T-bet-/-
B
Supplemental Figure 8: T-bet Expression in T cells from the T-betΔNCR+ILC mouse is Not Altered, Related to Figure 5.
A) Representative histogram showing intracellular staining for T-bet in NKp46+ and NKp46- CD3+ cells from WT, T-bet−/− and T-betΔNCR+ILC mice. B) Real-time PCR measuring the transcripts of Tbx21, Ifng and Gata3 in Th1 and Th2 in vitro differentiated cells from the spleen of WT, T-bet−/− and T-betΔNCR+ILC mice (n=3). Fold change expressed as mean ± SEM vs. T-betΔNCR+ILC.
Data are expressed as mean ± SEM and are representative of at least three independent experiments. ns: non-significant; ***p<0.001.

## Slide 9
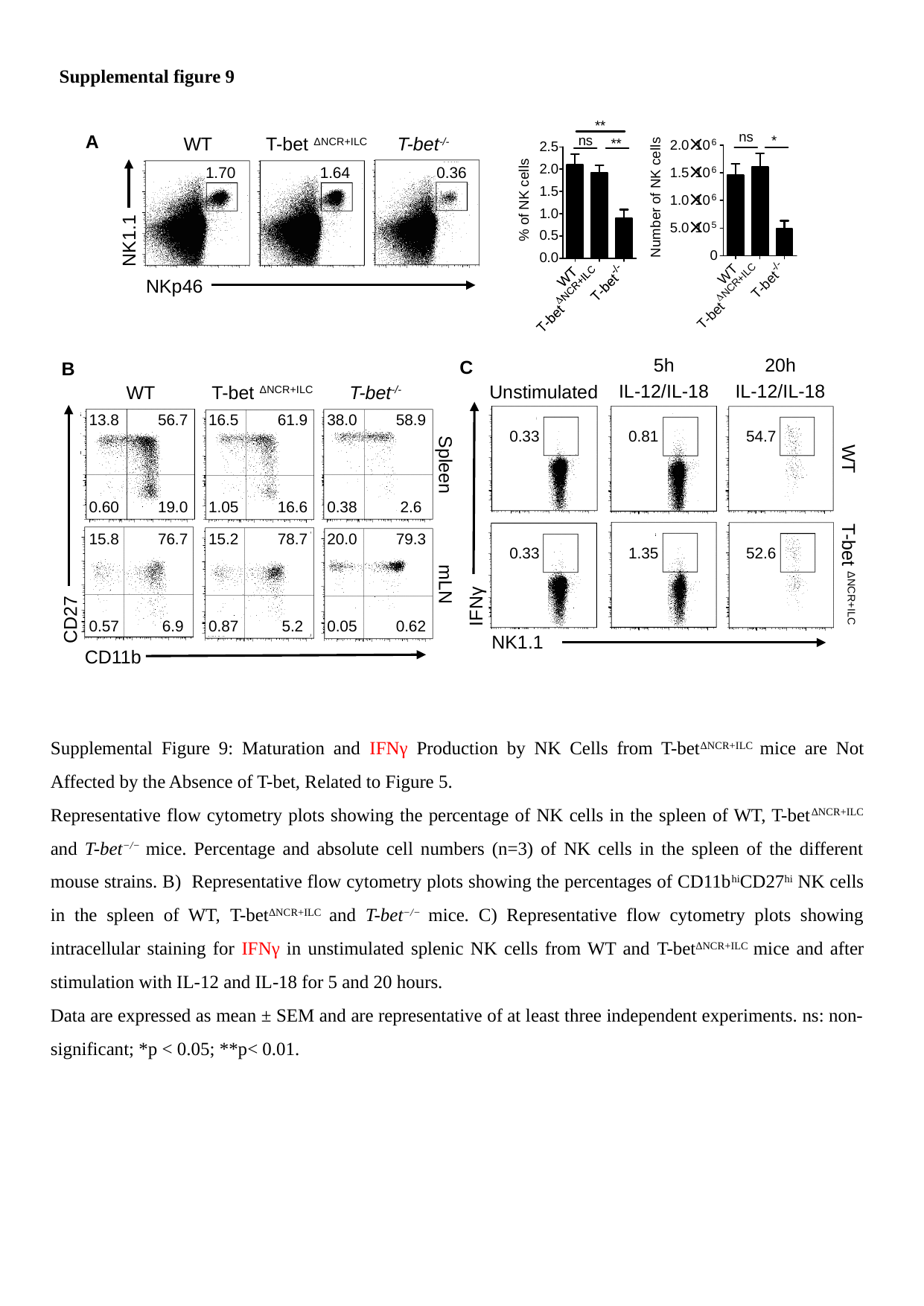

Supplemental figure 9
A
WT
T-bet ΔNCR+ILC
T-bet-/-
NK1.1
NKp46
1.70
1.64
0.36
5h
IL-12/IL-18
20h
IL-12/IL-18
Unstimulated
0.33
0.81
54.7
WT
0.33
1.35
52.6
T-bet ΔNCR+ILC
IFNγ
NK1.1
C
B
WT
T-bet ΔNCR+ILC
T-bet-/-
Spleen
mLN
CD27
CD11b
13.8
56.7
16.5
61.9
38.0
58.9
0.60
19.0
1.05
16.6
0.38
2.6
15.8
76.7
15.2
78.7
20.0
79.3
0.57
6.9
0.87
5.2
0.05
0.62
Supplemental Figure 9: Maturation and IFNγ Production by NK Cells from T-betΔNCR+ILC mice are Not Affected by the Absence of T-bet, Related to Figure 5.
Representative flow cytometry plots showing the percentage of NK cells in the spleen of WT, T-betΔNCR+ILC and T-bet−/− mice. Percentage and absolute cell numbers (n=3) of NK cells in the spleen of the different mouse strains. B) Representative flow cytometry plots showing the percentages of CD11bhiCD27hi NK cells in the spleen of WT, T-betΔNCR+ILC and T-bet−/− mice. C) Representative flow cytometry plots showing intracellular staining for IFNγ in unstimulated splenic NK cells from WT and T-betΔNCR+ILC mice and after stimulation with IL-12 and IL-18 for 5 and 20 hours.
Data are expressed as mean ± SEM and are representative of at least three independent experiments. ns: non-significant; *p < 0.05; **p< 0.01.

## Slide 10
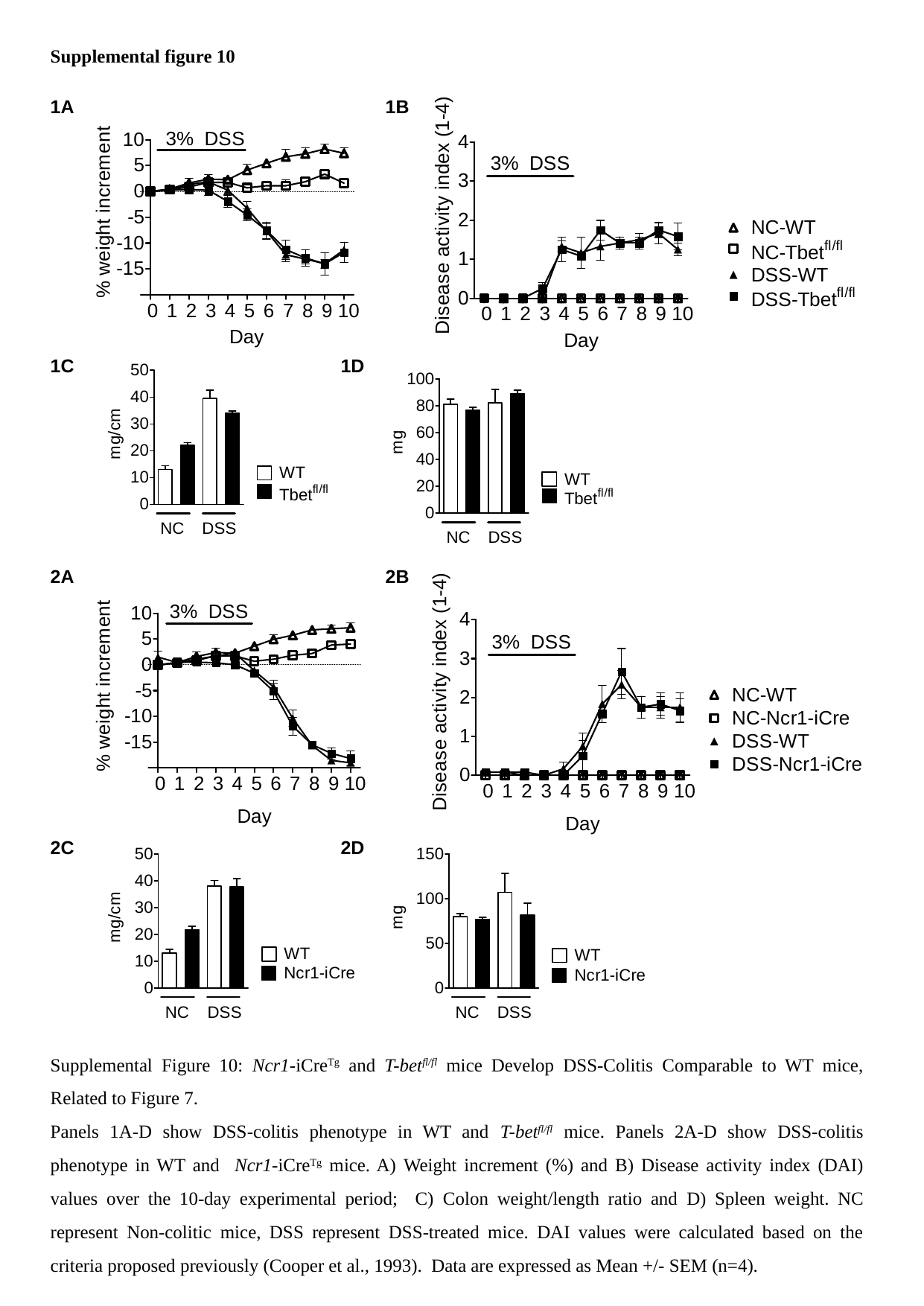

Supplemental figure 10
1A
1B
1C
1D
2A
2B
2D
2C
Supplemental Figure 10: Ncr1-iCreTg and T-betfl/fl mice Develop DSS-Colitis Comparable to WT mice, Related to Figure 7.
Panels 1A-D show DSS-colitis phenotype in WT and T-betfl/fl mice. Panels 2A-D show DSS-colitis phenotype in WT and Ncr1-iCreTg mice. A) Weight increment (%) and B) Disease activity index (DAI) values over the 10-day experimental period; C) Colon weight/length ratio and D) Spleen weight. NC represent Non-colitic mice, DSS represent DSS-treated mice. DAI values were calculated based on the criteria proposed previously (Cooper et al., 1993). Data are expressed as Mean +/- SEM (n=4).
